# Dynamic vulnerability revealed in the collapse of an Arctic tidewater glacier

**DOI:** 10.1038/s41598-019-41117-0

**Published:** 2019-04-03

**Authors:** Christopher Nuth, Adrien Gilbert, Andreas Köhler, Robert McNabb, Thomas Schellenberger, Heïdi Sevestre, Christian Weidle, Luc Girod, Adrian Luckman, Andreas Kääb

**Affiliations:** 1 Department of Geosciences, University of Oslo, Oslo, Norway; 2 University Centre in Svalbard (UNIS), Longyearbyen, Norway; 3 Institute of Geosciences, Kiel University, Kiel, Germany; 4 Department of Geography, College of Science, Swansea University, Swansea, UK

## Abstract

Glacier flow instabilities can rapidly increase sea level through enhanced ice discharge. Surge-type glacier accelerations often occur with a decadal to centennial cyclicity suggesting internal mechanisms responsible. Recently, many surging tidewater glaciers around the Arctic Barents Sea region question whether external forces such as climate can trigger dynamic instabilities. Here, we identify a mechanism in which climate change can instigate surges of Arctic tidewater glaciers. Using satellite and seismic remote sensing observations combined with three-dimensional thermo-mechanical modeling of the January 2009 collapse of the Nathorst Glacier System (NGS) in Svalbard, we show that an underlying condition for instability was basal freezing and associated friction increase under the glacier tongue. In contrast, continued basal sliding further upstream increased driving stresses until eventual and sudden till failure under the tongue. The instability propagated rapidly up-glacier, mobilizing the entire 450 km^2^ glacier basin over a few days as the till entered an unstable friction regime. Enhanced mass loss during and after the collapse (5–7 fold compared to pre-collapse mass losses) combined with regionally rising equilibrium line altitudes strongly limit mass replenishment of the glacier, suggesting irreversible consequences. Climate plays a paradoxical role as cold glacier thinning and retreat promote basal freezing which increases friction at the tongue by stabilizing an efficient basal drainage system. However, with some of the most intense atmospheric warming on Earth occurring in the Arctic, increased melt water can reduce till strength under tidewater glacier tongues to orchestrate a temporal clustering of surges at decadal timescales, such as those observed in Svalbard at the end of the Little Ice Age. Consequently, basal terminus freezing promotes a dynamic vulnerability to climate change that may be present in many Arctic tidewater glaciers.

## Introduction

Glacier flow instabilities can rapidly decrease total ice mass^1,2^ as opposed to the slower processes of accumulation and ablation. A key question is whether events resulting in rapid glacier dynamical change cause reversible or irreversible consequences in ice mass at decadal to millennial perspectives^3^. Glacier dynamic instabilities are associated with switches between slow and fast glacier flow, and may occur on ice streams^4^, after loss of ice shelves^5^, on tidewater^1^ and land terminating glaciers^6^ in response to sudden change in basal and/or frontal conditions. Recent observations suggest a continuum of glacier dynamic behavior^7^ with the most extreme cases when entire glaciers slide down valleys^8^.

Surging glaciers provide an important natural laboratory for understanding dynamic instabilities with pulsating glacier flow and intrinsically unstable conditions^6,9–12^. A glacier surge is defined by abnormally fast flow over a relatively short period (months to years)^13^, and glacier surges have been observed to occur in geographic clusters around the globe^14^. Controlling processes behind surge behavior are not fully understood^9,15^, particularly in relation to internal mechanisms and external forcing^14^. Suggested mechanisms for active surges include changes in the configuration of the basal drainage system^10^, interactions between till deformation and drainage efficiency^6^ or thermal control on basal conditions^12,16,17^.

Svalbard holds a prominent cluster of surge-type glaciers^14^. Situated at the tail end of the North Atlantic Current, climate in the Svalbard archipelago is highly sensitive to atmospheric and oceanic influences^18^, with some the most intense warming in the Arctic, +1 °C air temperature every 4 decades^19^. Current climate change impacts on Svalbard therefore serve a potential model for future changes in colder Arctic regions in which atmospheric warming may be delayed. The cold and dry climate with a mean annual air temperature of −6 °C over the past century^19^ is reflected in glaciers with lower mass turnover rates (i.e. the renewal of mass through a glacier) than other regions with high concentrations of surging glaciers such as in Alaska, Canada and the Himalayas. Most glaciers are polythermal, with a 100–200 m cold surface layer overlying warmer basal ice^20–22^, as common around the Arctic^23–25^.

**Figure 1:**
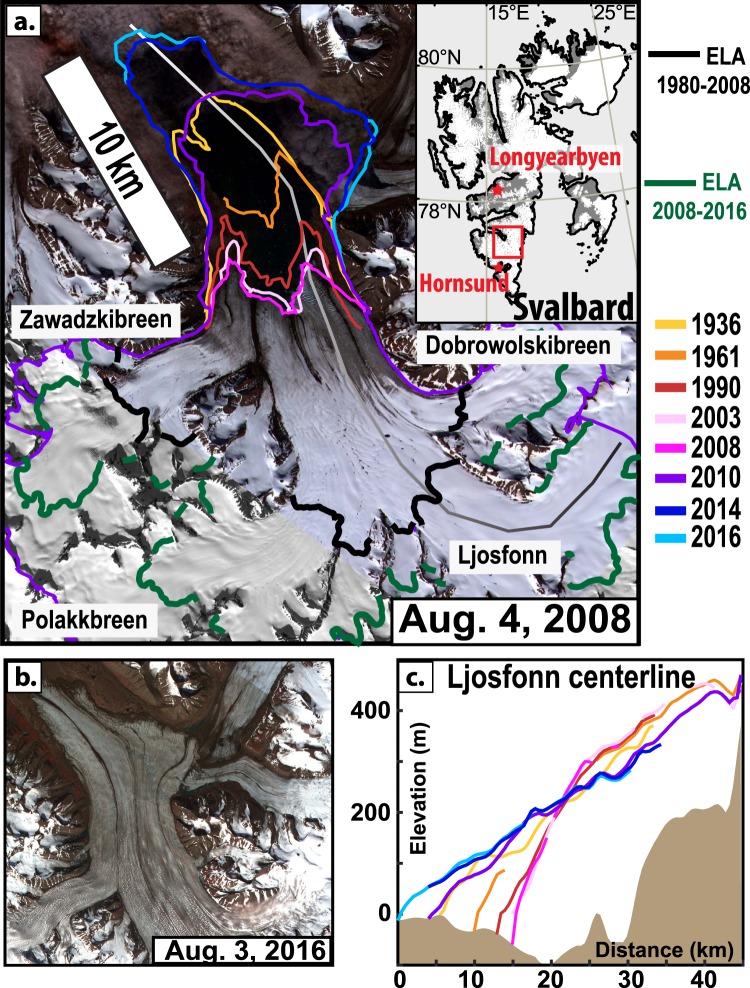
The Nathorst Glacier System (NGS) before and after its collapse. In south central Spitsbergen (**a**, inset), the NGS is observed from ASTER satellite imagery in 2008 (**a**) and 2016 (**b**). Centerline elevation profiles over the past 70 years (**c**) of the Ljosfonn tributary (gray to black line in (**a**)). Modeled Equilibrium Line Altitudes (ELA) from 1980–2008 and 2008–2016 are shown in (**a**) as thick black and green lines, respectively. Front positions (**a**) and elevation profiles (**c**) are synchronously color coded according to their year.

The Nathorst Glacier System (NGS) in south central Spitsbergen comprises four main tributary glaciers, covering 430 km^2^, that merge into a 5 km wide tidewater calving front (Fig. 1a). Submarine bathymetry reveals a surge in the mid to late 19th century^26^ that reached the extent of a surge ≈2700 years ago^27^. The glacier retreated 12 km from 1936 to 2008, exposing a large submarine esker imprinted on the sea-floor^26^ that suggests efficient subglacial drainage^28^. Radio echo soundings along a central flow line in spring 1980^29^ show internal reflection horizons at 150–200 m depth on the lower glacier^20^ that denote a cold-temperate transition surface (CTS)^30^ and thus temperate basal conditions below a significant cold ice layer.

## Observations

### Pre-Collapse

**Figure 2:**
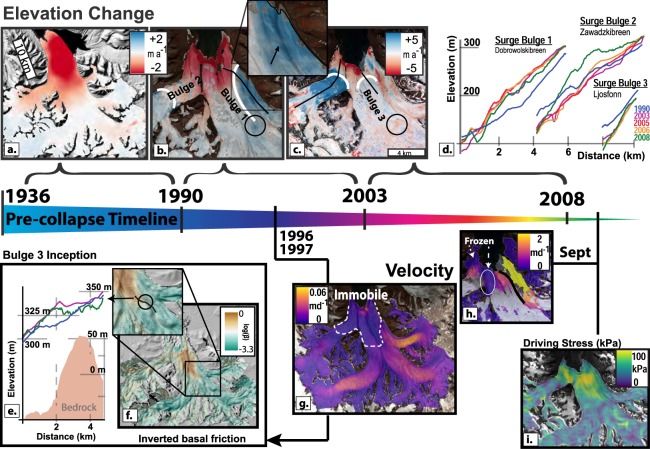
Pre-collapse observational time line. Glacier elevation changes from 1936 to 1990 (**a**), 1990 to 2003 (**b**) and 2003 to 2008 (**c**). Approximate bulge locations are shown by white lines. (**b**, inset) shows the un-moved medial moraine in relation to Bulge 1. In (**d**), centerline elevation profiles (shown as black lines in (**b**,**c**)) through time of the three surge bulges with colors represented by time. The black circle (in **b**,**c**) shows the anomalous thickening related to the inception of Bulge 3 (**e**), corresponding to the edge of the 1996/97 zone of high basal friction (**f**). Background 2010 DEM hillshade in (**f**) shows an imprint of the bedrock topography, transposed onto the post-collapse surface. The interferometric velocity field from the ERS 1/2 mission (**g**) used to invert basal friction in (**f**), Monthly velocity measured in late summer between ASTER and SPOT-5 orthoimages (**h**), a few months prior to collapse. Black lines denote a newly developed shear margin with the initiation of rapid flow of Dobrowolskibreen. Pre-collapse driving stress at the base of the glacier in 2008 is shown in (**i**).

Glacier surface displacements, or the lack thereof, measured from repeat satellite images, reveal the evolving NGS dynamics prior to the 2009 collapse. Interferometric processing of ERS 1/2 radar image pairs (1996/1997) shows a large region of the tongue with velocities lower than the measurement noise of 0.01 m d^−1^ (Fig. 2g, Supplementary Information). Combined with estimates of the glacier bed topography^31^, we inverted this velocity field to estimate the concurrent basal friction coefficient (Methods, Supplementary Information). High-friction conditions exist across the entire width of the outlet terminus (≈30 km^2^, Fig. 2f) which contrasts to lower friction elsewhere in NGS.

Digital Elevation Models (DEMs) provide geometrical timestamps that document the sequence of events that led to collapse. Surface bulges formed on two of the tributary glaciers, Dobrowolskibreen (Fig. 1; Bulge 1 in Fig. 2b,d) and Zawadzkibreen (Bulge 2), measurable between 1990 and 2003. While the Dobrowolskibreen bulge migration was finished by 2003 (Fig. 2b,d), the extent of a 10–20 m thick forebulge (Fig. 2b inset) coincides with the region of fast flow that eventually breached the terminus by 2008 (Fig. 2h). On the other hand, the Zawadzkibreen bulge migrated and thickened rapidly from 2006 to 2008 (Fig. 2b,d) which increased driving stresses eventually displacing the medial moraine dividing it from Polakkbreen (Supplementary Information).

**Figure 3:**
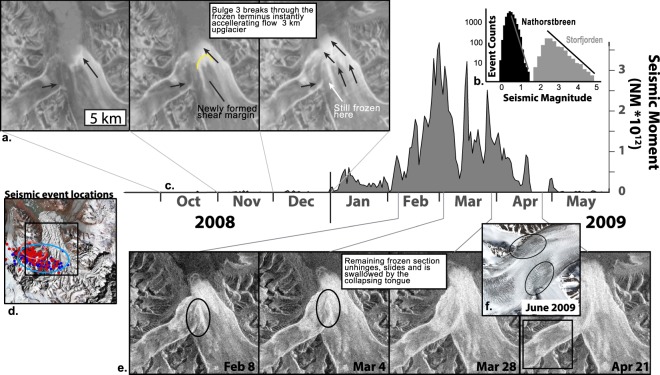
Observational timeline of glacier collapse. Heavily crevassed areas return high backscatter (bright) in winter radar images. Monthly compiled Envisat ASAR images (**a**) show the development of a new shear margin from Bulge 3 in the last 3 months of 2008. The magnitude distribution of NGS seismic events in relation to the 2008 Storfjord earthquake located about 50 km to the west is shown in (**b**). Seismic moment estimated from local magnitudes (**c**) define the timing of the collapse. Locations of the largest 96 seismic events shown with error ellipses in (**d**). Red/blue colors represent the locations and errors when determined from Longyearbyen/Hornsund (Fig. 1c), respectively. Radarsat-2 images every 24 days in the first four months of 2009 (**e**) show the mechanical destabilization of the remaining sticky spot in February. Seismic event locations over the central region of Zawadzkibreen (**d**) coincide with large rifts and damaged ice areas seen on a high resolution June 2009 image (**f**).

A third and much smaller bulge formed on Ljosfonn outside the Dobrowolskibreen confluence, apparent as a circular pattern of elevation increases (Fig. 2b,e) at the upstream margin of the high basal friction patch (Fig. 2f). This 1 km wide and 20 m high surface bump, which resembles subglacial lake filling^32^, progressed downstream (Supplementary Information) breaching the calving front by the end of 2008 (Fig. 3a). The development, timing, and force induced by these surge bulges increased driving stress on the tongue towards the limiting friction point of the till below, which by September 2008 reached 100 kPa as compared to 30–50 kPa over the rest of the glacier (Fig. 2i).

### Collapse

Finally in early 2009, the entire terminus of NGS rapidly destabilized leading to full collapse of the glacier system. Repeat Envisat ASAR and Radarsat-2 radar images capture the surface evolution during the dark Arctic winter (Fig. 3a,e). Heavily crevassed areas return high backscatter in winter radar images which in October 2008 were limited to the fast flow region generated by the Dobrowolskibreen surge. The crevassed area expanded along the calving front rapidly in November 2008 and a longitudinal strip of high backscatter extending from the terminus up-glacier coincides with a shear margin at the edge of the Ljosfonn surge bulge suggesting fast flow was initiated. The advance of the Ljosfonn terminus was forced westward by the advancing Dobrowolskibreen, towards the remaining frozen tongue of Zawadzkibreen (Fig. 3a). The remaining 5 km^2^ stagnant plug unhinged (Fig. 2h) causing rapid advance of the entire NGS terminus (>40 ± 1 m d^−1^) by early January 2009 (Fig. 3e), decaying to 30 ± 1 m d^−1^ by April (Supplementary Information).

The precise timing of collapse is detected up to 90 km away by ≈14,000 low magnitude seismic events from January to April 2009 (Fig. 3c), the strongest of which are clustered around a bedrock bump under Zawadzkibreen (Fig. 3d), and corresponding with large rifts and heavily damaged ice observed on July 7, 2009 (Fig. 3f). The magnitude distribution of events (Fig. 3b) exhibits earthquake-swarm like behavior and the temporal decay in event signal similarity combined with estimates of seismically recorded energy release suggest source propagation and/or no fault-healing (Supplementary Information) rather than a single sticky spot at the glacier base^33,34^. When the tongue suddenly slipped 6 km down the fjord, dramatic longitudinal extension ripped ice apart in areas already weakened by a relatively rapid bulge progression, thus producing the peculiar swarm of seismic signals (Supplementary Information).

### Post-Collapse

The NGS terminus slowed after the initial collapse, advancing only 5 km from summer 2009 to 2010 (7–10 m d^−1^) and another 5 km by 2016 (2–3 m d^−1^) (Supplementary Information). By this time, the glacier had nearly doubled its pre-collapse length. The rate of NGS mass loss in the years during (−2.33 ± 0.28 m w.eq. a^−1^ in 2008–2010) and after (−1.50 ± 0.35 m w.eq. a^−1^ in 2010–2014) the collapse is 500–900% greater than the long term 1936–2008 average of −0.25 ± 0.07 m w.eq. a^−1^ (Supplementary Information). We estimate the mass balance impact by the change in hypsometry caused from the instability using a surface mass balance model (Supplementary Information). In the current climate (2000–2016 average), the geometric change induced by the surge itself reduces the accumulation area by 35% which nearly doubles the negative surface mass balance (+84%). As the glacier was already unbalanced with climate before its collapse together with the 100–150 m rise in ELA over the past 2 decades, these changes are likely irreversible relative to the 2008 ice volume (Supplementary Information).

## Interpretation

**Figure 4:**
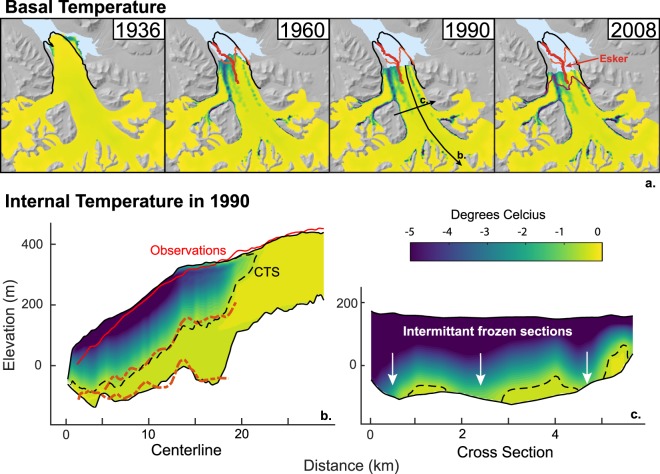
Thermo-mechanical model for the 70 year retreat prior to collapse Basal temperatures showing the expansion of frozen areas in green and blue at the terminus and along the moraines (**a**). The undisturbed subsea esker formed during the 1936–2008 retreat^26^ is shown as a red line. Front positions shown in 1936 (black), 1966 (orange) and 2008 (magenta). Centerline profile (**b**) of modeled Ljosfonn temperatures (color scale) and geometry (black lines) with the corresponding 1990 surface height observations (red line) and 1980 bed and CTS transition surfaces (red dashed lines^29^). The cross sectional profile (**c**) shows intermittent frozen sections along flow convergence areas.

Using a three-dimensional thermo-mechanical model^35^ forced by the long-term Svalbard meteorological time series from 1890^19^, we reconstruct the thermal and geometrical evolution of NGS since 1936 (Supplementary Information). Within a few decades following the previous surge, patches of ice along flow convergence regions on the tongue rapidly freeze (Fig. 4), such as under medial moraines where advection of warm ice is minimized. Furthermore, high friction must have been established over the entire tongue region at this time for the model to reproduce the 2008 glacier geometry, suggesting the friction anomaly results from basal freezing (Supplementary Information). With the observed extent of high friction (Fig. 2f) significantly larger than the modeled cold-based zone, we suggest that cold patches along flow convergence are sufficient to reduce the mean sliding speed and eventually stabilize an efficient drainage system out of the glacier through large channels in the sediment. Evidence is imprinted on the sea floor in the form of eskers^26^ that extend continuously from the 1936 terminus position to the 2008 terminus (Fig. 4). The capacity of those perennial channels, which evolved to handle the seasonal pulsations of meltwater flowing through the base, ensured low basal water pressure in the system. Effective pressure was large enough for till strength to support basal shear stress and therefore high friction over the tongue region even in temperate basal parts.

Cold patches provide a stabilizing positive feedback as reduced basal sliding promotes development of an efficient drainage system that further reduces sliding. Paradoxically, the potential for instability increases as continued basal sliding upstream allows build up of driving stresses on the tongue through surge bulges. Once pressure on the tongue reaches the ultimate shear strength of the till (limiting friction), basal friction enters an unstable regime controlled by the plastic rheology of the till^36,37^. The resulting change in back-stress up-glacier leads to a sudden increase in shear stress that propagated the instability through the entire glacier in only a few days. This rapidity, exemplified by the seismic record of ice rupture, suggests till failure. The NGS collapse illustrates thus how mechanisms leading to basal shear stress increase in the frontal regions of Arctic glaciers can start larger dynamic instabilities as long as till thickness is sufficient to enter an unstable state of friction (plastic behavior).

**Figure 5:**
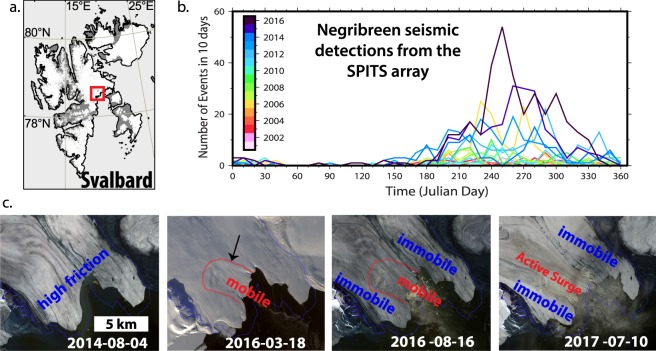
Negribreen destabilization. In March 2016, a section of the immobile terminus of Negribreen in Northeast Spitsbergen (**a**) seems to have collapsed, with rapid flow initiation by August 2016. Automatically detected and located seismic events (**b**) from the SPITS array in Longyearbyen (80 km away) show increased activity in late 2015. Satellite images from Landsat 8 and Sentinel 2 (**c**) show the break up of the high friction, stagnant Negribreen tongue. By July 2017, parts of the Negribreen basin are fully surging.

The sequence of events observed for Nathorstbreen is remarkably analogous to the surges in the Paulabreen catchment, though that glacier system did not collapse^38,39^ (Supplementary Information). In contrast to the simultaneous bulges in the NGS case, the non-synchronized timing of surge bulges limited driving stress increases on the tongue. In March 2016, Negribreen (Northeast Spitsbergen) destabilized with a frontal collapse signature on the terminus surrounded by stagnant regions easily visible in satellite images and corresponding to enhanced seismicity recorded in Longyearbyen (Fig. 5). Eskers imprinted in front of Paulabreen, in the sea floor in front of Negribreen, and in front of many other glaciers in Northeast Spitsbergen^40^, are formed from long term stabilized efficient drainage systems out of the glacier^28^, similar to Nathorstbreen. We suggest this is indicative of a dynamic vulnerability from basal freezing, which may or may not be exploited. Active surges or full collapses may or may not develop depending upon the glacier geometry, basal till characteristics, ice thickness, thermal conditions and water budget, among other factors. Other recent examples include Basin-3 and then Basin-2 of Austfonna, Svalbard^1^, Stonebreen^41^, an outlet basin of the Vavilov ice cap in Arctic Russia^2^, and many more over the past decade^42^. A common feature is that these glaciers are retreating and thinning in very cold climates and at the marine-land transition, often with ice-flow constrictions just upstream from the terminus. In this manner, dynamic instabilities could be explained, at least partially, by the mechanisms revealed here in detail for NGS.

## Conclusions

The NGS collapse shows that an underlying condition for this type of glacier flow instability is reaching an ultimate till shear strength under the tongue. We identify that a key process leading to such conditions was basal freezing of patches under the glacier tongue, a process that is dependent on climate. These cold patches allow stabilization of an efficient basal drainage system and thus high friction under the tongue, which in turn creates surface steepening and increase in driving stress. Paradoxically, glacier thinning and retreat increases potential for instability as reduction in both ice thickness and advection of warm ice from accumulation areas increase the possibility for basal freezing. Increased melt water production in a warmer climate may at some point override the stability provided by efficient basal drainage systems that, for example, have developed and evolved in a cooler world with less meltwater. This could increase basal water pressures and reduce till strength in the glacier tongue areas. Through these seemingly contrasting mechanisms of increased basal freezing and enhanced meltwater production, both imposed by atmospheric warming, changes in climate could therefore orchestrate a temporal clustering of surging on decadal timescales, providing a viable explanation for the rather large number of Svalbard tidewater glacier surge events at the end of the Little Ice Age^22,40^, and now also more recently^42^. The similarity between the mechanism here with the binge-purge oscillations described for ice stream behavior^43,44^, for example in Heinrich events, is fascinating as both are controlled by basal freezing. At the smaller scales of tidewater glaciers however, the influences of geometry, thickness, sediments and water production would lead to a diversity of responses and thus significant variability in the repeat frequency of surge events. Under current climate and projected change, the consequences of this tidewater glacier destabilization are irreversible as the currently rising equilibrium line altitude limits the accumulation needed to replenish the ice mass lost. With increasing air temperatures^45^, the thinning and retreat of tidewater glaciers in cold climates may promote conditions favorable for dynamic instabilities, such as the NGS collapse.

## Methods

### Seismology

Seismic broadband data were collected from the permanent single station at Hornsund (HSPB) and the Spitsbergen seismic array (SPITS) in Longyearbyen, which consists of 9 receivers with an aperture of 1 km. Event localization and autonomous signal detection was accomplished using the methods described by^46^. Event localization could be performed on 96 manually picked events, using broadband FK analysis on data from SPITS^47^ and three-component polarization analysis on HSPB^48^ between 2 and 10 Hz to determine the back-azimuths of seismic events. Back-azimuths and travel time data were inverted for epicenters using HYPOSAT^49^. The depth was fixed at the surface and the BARENTS3D regional velocity model was used^50–52^.

#### Event locations

Large epicentral distance and a low number of observing stations limits the spatial resolution of event localizations reflected by the scattering of epicenters^46^ and Fig. 3. Hence, spatial-temporal patterns inside the event cluster are not interpreted. Location uncertainty is estimated from standard deviation of back azimuth and distance-proportional S-P wave travel time difference measurements independently for HSPB and SPITS. Travel time difference (TD) is converted into distance (d) using the approximation:

1 $$d=TD\times vs\times vp/(vp-vs)$$

Seismic velocities (*νp*, *νs*) are varied within realistic ranges to incorporate uncertainties of the regional velocity model. Mean and standard deviation of back-azimuth and distance (from all events and all tested velocities) are used to draw the error ellipsoids in Fig. 3 (red: SPITS, blue: HSPB). Assessment of a possible bias due to an incorrect seismic velocity model or miss-classified seismic phases (i.e. *Pn* vs. *Pg*), showed that the position of the seismic cluster upstream NGS is well constrained and that a location at the terminus can be ruled out.

#### Automatic event detection

The strongest events represent only a small portion of the total seismicity emitted at NGS in early 2009. The more complete event catalog is obtained through single-station master event cross-correlation^46^ and array-based waveform correlation^53^. Since there is some degree of variety in the waveforms of located events, we select a number of 25 representative master events for cross-correlation detection. The first 8 (HSPB) and 22 seconds (SPITS) of all master events are cross-correlated with continuous data from 2008 to 2013 of station HSPB and stations of the SPITS array after band-pass filtering between 2 and 8 Hz. Detections are obtained by applying a threshold of 0.5 to the cross-correlation functions. Repeated detections resulting from different master events, different stations, or both correlation methods are rejected. In a second step, we perform beam-forming (FK analysis) between 2 and 8 Hz on a time window of SPITS data where the P and secondary arrivals are expected. Through autonomous measurements of back-azimuth, time difference between P and secondary arrivals, and apparent seismic velocity at maximum beam-power on all three wave-field components (vertical, radial, transverse), the origin of the corresponding signal at NGS is either confirmed or the detection is rejected^46^.

We perform a similar processing to obtain the time series of seismic events at Negribreen (Fig. 5), which are most likely calving events. However, instead of waveform cross-correlation, events are detected at SPITS using a STA/LTA trigger on array beams pointing into the direction of Negribreen. The resulting detections are then FK-processed in the same way as the NGS events to select signals with backazimuth and S-P travel time corresponding to the Negribreen area.

#### Seismic moment release

The observed seismicity is most likely only a small fraction of the (potential) total energy release because of the high detection threshold due to the long epicentral distance and/or aseismic deformation. We determined the seismic moment (*M*_0_) from the local magnitude (*ML*) using the empirical relation^54^:

2 $${M}_{0}={10}^{(ML+\mathrm{6)}\times 1.5}$$

The seismic moment is related to the fault or slip area (*A*), and the slip distance (*d*) through the relation

3 $${M}_{0}={\mu }_{ice}\times A\times D$$

where *μ*_*ice*_ represents the bed rigidity (shear modulus of ice) with typical values between 2.3 and 3.55 GPa^55^.

### Glacier elevation change and geodetic mass balance

The geometric evolution of the NGS system is derived by comparing historic, aerial and satellite derived DEMS spanning from 1936 to 2016. The 1936 data are contours from oblique aerial photography which have an accuracy of 15–20 meters^56,57^. The 1961 and 1990 DEMs (20 m horizontal resolution) are derived from modern photogrammetric methods on vertical aerial imagery^58^. These elevation products are referenced to a local geoid and have an accuracy of about 5 meters. All three of these national DEM products do not fully cover the NGS system. For example, the 1936 contour data is severely suspect at upper elevations do to lack of visual contrast in the far view of the oblique images. The 1961 DEM only covers a portion of the tongue and the upper elevations of Doborowolskibreen and Ljosfonn while the 1990 DEM is missing the upper part of Doborowolskibreen and Ljosfonn. All DEMs after 1990 are derived from satellite products and are referenced to WGS84 ellipsoid heights. We use state-of-the-art processing (MMASTER)^59^ to convert ASTER near-infrared stereo images (2003, 2005, 2006, 2008, 2010, 2014, 2016) into DEMs. By removing cross-track vibration perturbations not captured in metadata and along-track shaking artifacts when comparing DEMs, local accuracy of MMASTER products (±5 m) improved 4-fold compared to the legacy AST14DMO product (±20 m)^59^. Another 2008 DEM (40 m resolution) is a SPOT5-HRS product generated during the IPY as part of the IPY-SPIRIT project^60^. Last, we use the TanDEM-X Intermediate DEM^61^, re-sampled (block median) from 12 m to 36 m resolution, which is derived from 2 overpasses on December 14 and 19, 2010.

To compare, all products are first set into the WGS84 reference frame and datum, UTM projection 33. For the earlier DEMs, heights are converted to ellipsoid heights using the EGM2008 model. Using stable terrain (surrounding the glaciers), we co-register^62^ all products to both the TanDEM IDEM and the 1990 DEM. Triangulation of the co-registration vectors of all DEMs with these two reference DEMs reveals the horizontal and vertical accuracy of our geometric time-series (see, for example)^63^ to better than 5 and 1 m, respectively. The standard deviation of differences on stable terrain, after outlier removal, provide the local precision of differences to be on the order of 10–13 m for the ASTERS, and 5 m for the SPOT, TanDEM and aerial DEMs. These numbers provide the baseline for error estimates at a 67% confidence interval.

Volume changes between the epochs are derived from elevation changes following a hypsometric approach^64^.

4 $${\rm{\Delta }}V={\rm{\Sigma }}\,[{\rm{\Delta }}H(z)\times A(z)]$$

We estimate the volume change for each of the four tributaries separately, and then sum for the entire NGS system. Changes are binned every 50 meters (*z*) and averaged (Δ*H*). Missing areas are interpolated using up to 3rd order polynomials of the elevation differences with elevation. The hypsometry (*A*) is updated for each epoch using the DEM with the largest glacier area, and missing areas are filled in through spring metaphor in-filling techniques on the DEMs. Geodetic mass balance is derived by dividing the volume change by the average area in the epoch and multiplying by a density conversion factor of 0.85^65^. Errors are quantified using the same equations with the elevation error estimates above taking into account a 500 m spatial correlation^66^, assuming a 10% area error and density conversion error of 0.1 kg m^−3^, summed in quadrature.

### Glacier velocity

A precise glacier wide velocity map of NGS was acquired by differential Interferometric SAR (D-InSAR) performed on two ERS-1/ERS-2 tandem pairs. The ascending (ASC) pair was acquired on 17/18 December 1997, the descending (DESC) on 21/22 March 1996. From both pairs we derived line-of-sight velocities, which were then combined to a 2D-velocity map. The D-InSAR processing was performed using the GAMMA Remote Sensing software suite. The combination of ASC and DESC displacement maps and the accuracy assessment was performed in MATLAB. The processing consists of the following steps:

- RAW data to GAMMA single look complex (SLC) format
- Offset estimation between the two SLC images
- Preparation of DEM for 2-pass interferometry including sub-setting to joint coverage of ASC/DESC pair
- Computation of differential interferograms (ASC/DESC):
- Co-registration of DEM to MLI
- Simulation of topographic phase and differential interferogram
- Baseline refinement
- Unwrapping
- Calculation of displacement maps (ASC/DESC) in line-of-sight
- Geocoding
- Combination of ASC and DESC maps to 2D velocity map (MATLAB)
- Calculation of bedrock displacement (MATLAB) as measure of accuracy.

The accuracy was measured by extracting displacements of non-moving features. Velocities were extracted across a point grid of 1000 m spacing excluding those points which were not on stable terrain (glaciers, fjords, etc.). We found a median of 4.8 mm and a standard deviation of 6.3 mm for the ASC velocity map, and 2.0 mm and 6.6 mm for the DESC, respectively. The combined map has a median of 6.3 mm and a standard deviation of 7.0 mm.

Additional pre-collapse velocity fields (Fig. 2, Supplementary Information) were generated using optical image matching techniques^67^ on sequential Landsat, ASTER and SPOT orthoimages from 2003 to 2008. Images are separated by one year, except for the August 2008 velocity which compares an ASTER and SPOT image taken a month apart. Target window sizes are larger (500 m) to accurately capture persistent ablation area features with different surface conditions common in the annual time separation. After co-registration, off-glacier displacements are normally distributed with standard deviations of 10 meters (0.03 m d^−1^ for an annual image pair).

After the collapse, surface velocity maps were extracted from repeat Radarsat-2 Wide Mode (RS-2 W) data by means of SAR offset and speckle tracking. The ground resolution of the RS-2 W data is 19.3 × 5.0 m in range and azimuth, respectively. Displacements are estimated by the cross-correlation between two consecutive acquisitions^68^. Offsets were measured at 100 m spacing (5 × 20 pixel) with a search window of 800 m (40 × 160 pixel). The accuracy of this method was estimated previously in^69^. Displacements of GPS stations on Kronebreen, NW-Spitsbergen, compared to RS-2 W-based maps showed an R2 = 0.90 and a RMSE = 0.17 m d^−1^.

### Glacier Evolution Modelling

#### Surface mass balance model

Net surface mass balance is modelled using a degree-day method and is determined by:

5 $$M=Accu+R-Melt$$

where *M* is the net surface mass balance (m w.eq. yr^−1^), *Accu* is the total snow accumulation (m w.eq. yr^−1^), *R* is the rate of superimposed ice formation (m w.eq. yr^−1^) and *Melt* is the annual melting (m w.eq. yr^−1^).

Annual mass balance is directly computed from annual accumulation and annual melt using cumulative Positive Degree Days (*PDD*) over the year. Snow accumulation is calculated as a function of elevation (*z*):

6 $$Accu=\sum _{i=1}^{365}\,(\begin{array}{ll}{P}_{ref}(i)\times (1.0+(z-z{p}_{ref})\frac{{\rm{d}}p}{{\rm{d}}z}), & {\rm{if}}\,{P}_{ref}(i) > 0\,\& \,{T}_{air}(i,z) < {T}_{snow}\\ \mathrm{0,} & {\rm{if}}\,{P}_{ref}(i)=0\,\& \,{T}_{air}(i,z)\ge {T}_{snow}\end{array}$$

where *P*_*ref*_ is the daily precipitation rate (m yr^−1^) at the elevation zp_*ref*_ (m a.s.l), $\frac{{\rm{d}}p}{{\rm{d}}z}$ is the precipitation lapse rate (% *m*^−1^), *T*_*air*_ is the air temperature (K), *z* is the elevation (m a.s.l.) and *T*_*snow*_ a temperature threshold that distinguishes between snow and rain.

We formulate the degree-day factor as a function of surface type (ice or snow) by taking into account initial snow cover, superimposed ice formation, and total melt. The spatial distribution of the degree-day factor is recomputed each year as a function of the degree-day factors for snow and ice. This procedure allows us to take into account snow-albedo feedbacks. Annual melt is calculated using a degree-day model with the degree-day factor *f*_*m*_ (m w.eq. K^−1^ yr^−1^):

7 $$Melt=PDD\times {f}_{m}$$

The number of positive degree-days per year (*PDD*) is defined as a function of elevation (*z*):

8 $$PDD=\sum _{i\mathrm{=1}}^{365}{d}_{PDD}(i)$$

with

9 $${d}_{PDD}(i)=\{\begin{array}{ll}{T}_{ref}(i)+{\rm{d}}T/{\rm{d}}z(z-{z}_{ref}), & {\rm{if}}\,{T}_{ref}(i)+{\rm{d}}T/{\rm{d}}z(z-{z}_{ref}) > {T}_{m}\\ \mathrm{0,} & {\rm{if}}\,{T}_{ref}(i)+{\rm{d}}T/{\rm{d}}z(z-{z}_{ref})\le {T}_{m}\end{array}$$

where *T*_*ref*_ (K) is the mean daily air temperatures at the elevation *Z*_*ref*_ (m a.s.l.), d*T*/d*z* is the temperature lapse rate (K m^−1^) and *T*_*m*_ is a threshold temperature (K) above which melt occurs.

Each year, the degree-day factor is calculated at each grid point by first computing the ratio *r*_*s*/*m*_ between accumulated snow (snow precipitation minus snow lost by superimposed ice formation) and total melt, and assuming *f*_*m*_ = *f*_*snow*_:

10 $${r}_{s/m}=\frac{Accu-R(1+\frac{{\rho }_{w}}{\mathrm{(1}-{\rho }_{s}\,/\,{\rho }_{i}){\rho }_{s}})}{PDD\times {f}_{snow}}$$

We then have:

11 $${f}_{m}=\{\begin{array}{ll}{f}_{snow}, & {\rm{if}}\,{r}_{s/m}\ge 1\\ {f}_{ice}-({f}_{ice}-{f}_{snow})\times {r}_{s/m}, & {\rm{if}}\,{r}_{s/m} < 1\end{array}$$

where *f*_*snow*_ is the degree-day factor for snow and *f*_*ice*_ that for ice. Details on the calculation of the refreezing term *R* can be found in^35^.

We calibrated the mass balance model from geodetic volume change over the period 1990–2003, which is little affected by calving. We therefore assumed that the volume change is due to surface mass balance only during this period. We use both total volume change and individual pixel elevation changes in the immobile terminus area where we assumed that elevation change corresponds to surface mass balance. Surface elevations to compute mass balance are calculated by linearly interpolating through the DEM timeseries (1936, 1990, 2003, 2008, 2010 and 2014). We use the air temperature time-series from Longyearbyen Airport meteorological station and assume a constant precipitation rate. We chose to fix all parameters according to literature and only tuned precipitation lapse rate and melting parameters. By using both total mass balance and surface melting in the terminus area, a strong constrain on both degree day factors (snow and ice) is provided by remote sensing data alone. The values of the parameters used in the mass-balance model can be found in the Supplementary Information.

Temperature measurements at Longyearbyen Airport before 1948 are available in a monthly average only. In order to extend the mass balance reconstruction back to 1899 we computed the melting following the same approach but using the monthly temperature anomaly rather than daily. We computed the monthly temperature threshold and melting factor by matching mean mass balance obtained from the monthly method and from the standard daily method over the period 1948–2016. We found that the monthly approach provides a good approximation of the modelled mass balance with daily temperature (Supplementary Information).

#### Thermo-mechanical modelling

The thermo-mechanical model used in this study is presented in detail in^70^. It solves the Stokes equations coupled with an enthalpy approach for solving energy conservation and including water percolation into the firn and refreezing. Changes in the glacier geometry are computed using a free surface equation. We adopt a pure viscous isotropic ice rheology following Glens flow law. The model is solved using the finite-element software Elmer/ice^71^. We adopt a linear friction law as a basal boundary condition for the Stokes equation that reads:

12 $${\tau }_{b}=\beta {u}_{s}$$

where ${\tau }_{b}$ is the basal drag (MPa), *β* the sliding coefficient (MPa yr m^−1^) and *u*_*s*_ the sliding speed (m yr^−1^).

In order to take into account water percolation and refreezing within the firn, we follow the approach by Gilbert *et al*.^72^, in this case using a 6-month time step. Time-dependent surface boundary conditions are therefore updated every 6 months for the enthalpy equation using:

13 $$H(T,\omega )={H}_{s}({T}_{s})\pm {\rm{\Delta }}{H}_{s}$$

where *H* is the enthalpy (J kg^−1^), *T* the temperature (K), *ω* the fraction of liquid water content, *T*_*s*_ is the annual mean surface temperature (K) and Δ*H*_*s*_ the mean surface enthalpy variation between two half years (J kg^−1^). The latent heat from annual surface melting (computed from the mass balance model) is released during summer as a function of firn density and temperature. We assume that water cannot percolate in pure ice and that it refreezes in the first cold layer.

The density is estimated from firn thickness *H*_*firn*_ (m w. eq.) which is computed as:

14 $${H}_{firn}(t+dt)=\{\begin{array}{ll}{H}_{firn}(t)+MB\times dt-{H}_{firn}\times adt & \\ 0 & {\rm{if}}\,{H}_{firn}(t\mathrm{) < 0}\end{array}$$

where *a* is a densification rate parameter (yr^−1^) and *dt* the timestep (yr). The density profile *ρ* (z) is then computed assuming a linear profile until *ρ* reaches the density of ice *ρ*_*ice*_:

15 $$\rho (z)={\rho }_{0}+\frac{{\rho }_{ice}-{\rho }_{0}}{{z}_{s}-{z}_{ice}}({z}_{s}-z)$$

where *ρ* is the density (kg m^3^); *ρ*_0_ is the surface density, *ρ*_*w*_ is the water density, *z* the vertical coordinate (m) and *z*_*ice*_ the coordinate of the firn/ice transition (m). From mass conservation, *z*_*ice*_ has to satisfy:

16 $${\int }_{{z}_{ice}}^{{z}_{s}}\,({\rho }_{0}+\frac{{\rho }_{ice}-{\rho }_{0}}{{z}_{s}-{z}_{ice}}({z}_{s}-z)){\rm{d}}z={\rho }_{w}{H}_{firn}$$

Solving Eq. 16 leads to the following expression for the density profile:

17 $$\rho (y)={\rho }_{0}+\frac{{\rho }_{ice}^{2}-{\rho }_{0}^{2}}{2{\rho }_{w}{H}_{firn}}({z}_{s}-y)$$

The bedrock topography was provided by J. Fürst (see^31^, Supplementary Information). Horizontal mesh resolution is about 300 m with 20 vertical layers and run on 10 partitions for parallel computing.

#### Modeling thermal regime

Solving percolation and refreezing in the firn needs a transient approach for solving the temperature field even at steady state, which require significant computing time in 3D. Therefore, we first run the thermo-mechanical model on a 2D flow line along the Polakkbreen branch of NGS assuming constant basal friction coefficient and mass balance (1899–1960 average). High vertical mesh resolution is adopted close to the surface (<2 m) to properly solve surface processes. We run the model starting with a uniform temperature field until steady state thermal structure is reached. This provides a relationship between 10m-depth temperature (T_10*m*_), surface temperature (T_*surf*_), firn thickness (H_*firn*_) and surface melting in order to parametrize percolation and refreezing in the 3D model. Results shows that in these climatic conditions, 10m-depth temperature depends only on surface temperature and firn thickness since the amount of melting is never a limiting factor to produce temperate firn. The relationship can be parametrized as follows (Supplementary Information):

18 $${T}_{10m}=(\begin{array}{ll}{T}_{surf}+\mathrm{3.832(}{H}_{firn}{)}^{0.45}, & {\rm{if}}\,{T}_{10m} < 273.15\\ \mathrm{273.15,} & {\rm{if}}\,{T}_{10m}\ge 273.15\end{array}$$

This relationship is not significantly influenced by ice dynamics as it relies mainly on surface processes. Thus, the temperature structure in 3D can now be solved by only imposing a Dirichlet surface boundary condition set to the estimated 10m-depth temperature from firn thickness and surface temperature (Supplementary Information) without explicitly solving percolation and refreezing.

#### Basal friction inversion from the 1996 InSAR velocities

Basal friction coefficient (*β*) is inverted using a control inverse method to minimize a cost function defined from the misfit with measured surface data and a regularization term^71,73^. The inversion is done using the surface topography measured in 1990 after a 5-years run of free surface relaxation. The steady state temperature field is solved at each time step during the inversion in order to update ice viscosity according to ice temperature.

## Supplementary information

Supplementary Information: Dynamic vulnerability revealed in the collapse of an Arctic tidewater glacier

Video 1: Nathorstbreen collapse as seen from monthly compiled ESA Envisat images 

## Data Availability

A data package containing glacier velocity, geometric surface evolution, seismic record and modelling results is available through the NIRD Data archive at 10.11582/2019.00004. Most of the original data used in this study are available free of charge from various agencies. Radarsat-2 data are commercial. TanDEM-X Intermediate Products require permissions but scientific access schemes exist through DLR. The SPOT-5 SPIRIT data is available at the Theia Land Data Center through CNES. Landsat and ASTER imagery can be found at NASA Earth Data Search through EOSDIS. Sentinel-2 can be downloaded through Copernicus Sentinel Hub. Envisat is available through ESA. ORFEUS and EIDA (www.orfeus-eu.org) can be used for access to seismic waveforms and related metadata from Svalbard.
