# Supplementary material for: Dynamic vulnerability revealed in the collapse of an Arctic tidewater glacier
Source: Sci Rep. 2019 Apr 3;9:5541. doi: 10.1038/s41598-019-41117-0 (PMC6447555; doi:10.1038/s41598-019-41117-0)
Supplement: Supplementary file 1 — Supplementary Information: Dynamic vulnerability revealed in the collapse of an Arctic tidewater glacier [file 41598_2019_41117_MOESM1_ESM.pdf]

Supplementary Information:
Dynamic vulnerability revealed in the collapse of an Arctic tidewater glacier

Christopher Nuth[1,*], Adrien Gilbert[1], Andreas Köhler[1], Robert McNabb[1], Thomas Schellenberger[1], Heïdi Sevestre[2], Christian Weidle[3], Luc Girod[1], Adrian Luckman[4,2] and Andreas Kääb[1]

[1]Department of Geosciences, University of Oslo, Norway
[2]University Centre in Svalbard (UNIS), Longyearbyen, Norway
[3]Institute of Geosciences, Kiel University, Kiel, Germany
[4]Department of Geography, College of Science, Swansea University, UK
[*]Corresponding author: christopher.nuth@geo.uio.no

Contents

1 Data **2**

2 Seismology **4**
2.1 Event Magnitude Distribution 4
2.2 Event Similarity 4
2.3 Interpretation 6

3 Remote Sensing Analysis **7**
3.1 Radar backscatter 7
3.2 Geometric Observations and Interpretations 7

4 Glacier Evolution Modelling **16**
4.1 Model Parameters and Methods figures 16
4.2 Surface mass balance 18
4.3 Thermal regime and glacier geometry 21

1 Data

To unravel the series of events, we combine remote sensing data from over 10 different optical and radar satellite sensors with regional passive seismic records. Seismic events recorded at regional distances (> 50 km) signify the timing of the collapse over a few months from January to May 2009. State-of-the-art processing methods applied to antiquated ASTER optical stereo imagery [1] from 2003 to 2014 resolve topography to better than 5 m (30% of the pixel size) allowing temporal derivatives of topography to document the slow surge progressions of two of the tributaries. ERS1/2 interferometry from the 1990s and sequential optical orthoimages in the 2000s enable mapping of the stagnant tongue and evolutions of it prior to destabilization. Additional DEMs generated from 2008 SPOT5-HRS optical stereo images [2] and through interferometric processing of 2010 TanDEM-X radar imagery [3] constrain changes during the collapse event. Sequential ASAR C-band radar images from the deceased ENVISAT satellite and Radarsat-2 images provide the mechanical details of the collapse during the polar night. Last, offset and speckle tracking on sequential Radarsat-2 wide-beam imagery [4] detail the post-collapse velocity history (2009-2013). Finally, surface mass balance and thermo-mechanical modeling of the glacier evolution over the past 70 years provide the process based interpretations verified by our remote sensing observations.

Table S1: Remote sensing data sets used. MMASTER products are those described in [5]. The IPY-SPIRIT product are described in [2].

Year	Date	Aerial	Method / product	SENSOR	Resolution	Data ID
			**NATHORTSTBREEN**			
1936	Summer	Aerial	Topographic Contours	Camera	50 m	
1961	Summer	Aerial	Photogrammetric DEM	Camera	20 m	
1990	Summer	Aerial	Photogrammetric DEM	Camera	20 m	
1996	Mar 21,22	Satellite	Interferometry	ERS-1/2		
1997	Dec 17,18	Satellite	Interferometry	ERS-1/2		
2000	July 25	Satellite	Orthoimage	Landsat 7	15 m	LE72130042000207KIS00
2000	Aug 17	Satellite	Orthoimage	Landsat 7	15 m	LE72080052000236KIS00
2001	June 23	Satellite	Orthoimage	Landsat 7	15 m	LE72080052001174EDC00
2002	June22	Satellite	Orthoimage	Landsat 7	15 m	LE72120052002173EDC00
2002	July 12	Satellite	Orthoimage	Landsat 7	15 m	LE72080052002193EDC00
2003	July 24	Satellite	MMASTER DEM	ASTER	30 m	AST_L1A_00307242003113830_20161205045435_4078
2005	July 23	Satellite	MMASTER DEM	ASTER	30 m	AST_L1A_00307232005121537_20161205045435_4095
2006	July 23	Satellite	MMASTER DEM	ASTER	30 m	AST_L1A_00307232006114522_20161205045455_4274
2008	Aug 04	Satellite	MMASTER DEM	ASTER	30 m	AST_L1A_00308042008115156_20161205045445_4228
2008	Sep 01	Satellite	IPY-SPIRIT DEM	SPOT	40 m	SPI08_025_Svalbard
2008	Oct-Dec	Satellite	Radar Image	Envisat-ASAR	150	
2009	June 21	Satellite	Orthoimage	Landsat 5	15 m	LT52080052009172KIS00
2009-2013	24 Day repeat	Satellite	Radar Image	RADARSAT-2	20 x 5 m	
2010	Aug 10	Satellite	MMASTER DEM	ASTER	30 m	AST_L1A_00308102010115121_20161205045445_4219
2010	Dec 14, 19	Satellite	Intermediate DEM	Tandem	12 m	IDEM_30_N77E016
2014	July 22	Satellite	MMASTER DEM	ASTER	30 m	AST_L1A_00307222014113925_20161205045445_4236
2016	Aug 03	Satellite	MMASTER DEM	ASTER	30 m	AST_L1A_00308032016114627_20161205045455_4287
			**NEGRIBREEN**			
2014	Aug 4	Satellite	Orthoimage	Landsat 8	30 m	LC08_L1TP_210004_20140804_20170420_01_T1
2016	Mar 18	Satellite	Orthoimage	Landsat 8	30 m	LC08_L1TP_210004_20160318_20170328_01_T2
2016	Aug 16	Satellite	Orthoimage	Landsat 8	30 m	LC08_L1TP_211004_20160816_20170322_01_T1
2017	Jul 10	Satellite	Orthoimage	Sentinel 2	10 m	S2B_MSIL1C_20170710T122649_N0205_R052_T33XWH_20170710T122652

2 Seismology

2.1 Event Magnitude Distribution

Local seismic magnitudes are estimated for all detections using the maximum displacement amplitude measured on SPA0 (SPITS array station). We derive parameters for a local magnitude-amplitude relation using SPA0 amplitudes of regional tectonic earthquakes East of Spitsbergen [6]. The magnitude-frequency distribution of all events does not follow a classic Gutenberg-Richter law (power-law distributed seismic amplitudes) (Fig. 3, Main text). After testing different models, we find that the amplitude distribution is better explained by an exponential or log-normal decay towards higher magnitudes than a power-law (statistical power-law test, after [7]). For comparison, the Storfjorden earthquake aftershock sequence, which occurred 85 km east-southeast of NGS [6], clearly follows a Gutenberg-Richter law with an exponent (b value) closer to 1 as expected for tectonic seismicity (Fig. 3, Main text), and the statistical test rejects an exponential decay. Forcing a power-law fit to the NGS events results in an unusually high b value of 2.9 (Fig. 3, Main text). High b-values up to 3.0 have been reported for earthquakes swarms in volcanic regions where the crust is weakened and cannot accumulate high stress because of the presence of magmatic fluids [8, 9]. Similar arguments could be made for fracturing in ice. In fact, a wide range of b-values between 0.5 and 3.5 have been reported in cryo-seismological studies [10]. Furthermore, deviations from the Gutenberg-Richter-law were found, for example a more gauss-like magnitude distribution for repeating stick-split ice-quakes in Antarctica [11, 12]. We do not have indication that the NGS events occur at a single asperity (see above) and cannot resolve the seismic source mechanism. However, previous studies suggest that our observed magnitude distribution and even the b-value (forced fit) are physically reasonable in a cryospheric environment.

2.2 Event Similarity

The similarity between master events and their detections in Longyearbyen and Hornsund is highest a few days before and after the master event occurs (correlation coefficient >0.8)

and decreases continuously in the time period before and after (Fig. S2). This observation may indicate an increasingly damaged glacier area that alters seismic wave propagation near the source and/or that seismic sources propagate and do not occur again at exactly the same spot damaged previously (no fault-healing). However, this propagation is not resolved by any significant trends in S-P travel-time differences and back-azimuths over time (see above). If the seismic source would be a single sticky spot (asperity) at the base of the glacier with direct coupling to the solid earth, we would expect to observe higher correlation (>0.9) over a longer time period and a more regular recurrence interval [11, 12]. Furthermore, many more events with lower correlation are detected at the day of the particular master event. Hence, activity is most likely spread in the source area upstream Zawadzkibreen (within error ellipse in Fig. S4).

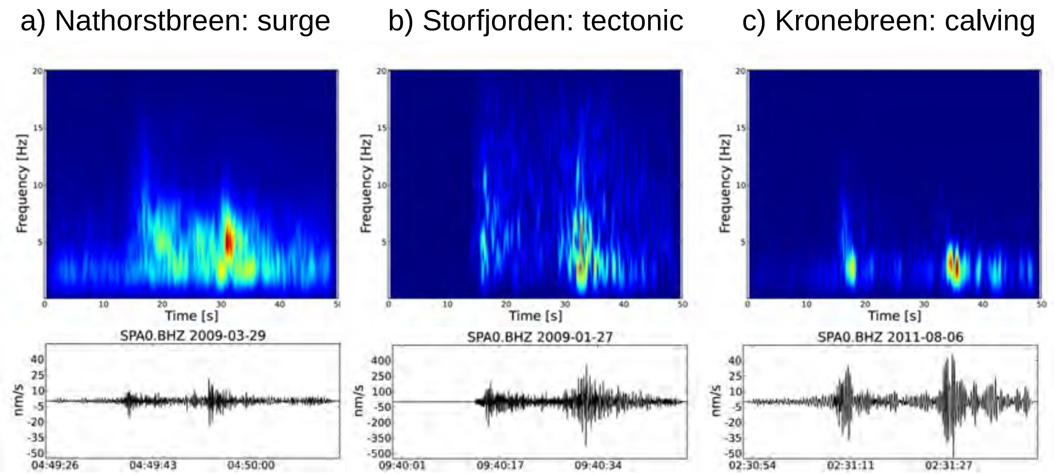

Figure S1: **Spectrograms and waveforms of the NGS collapse in relation to other dominant signals recorded in Svalbard at similar distances**. A stack of 96 signals from the NGS collapse [a] as compared to the 2008 tectonic earthquake 60 km to the east of NGS in Storfjorden[b], and glacier calving at Kronebreen, northwest Svalbard [c]

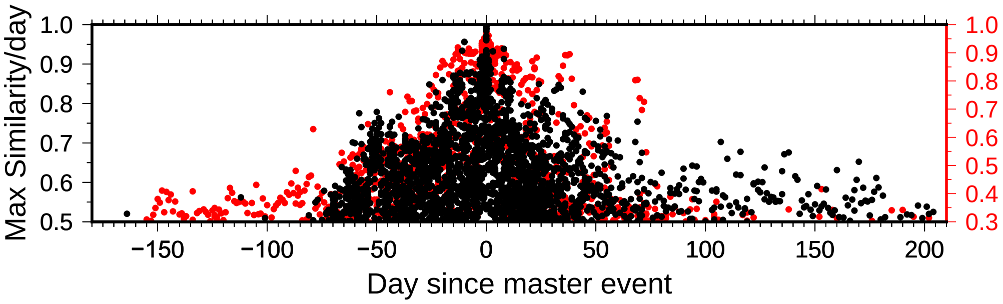

Figure S2: **Temporal decay in seismic signal correlation from NGS master events**. The cross correlation between selected master events and detected events decays with the temporal separation to the master event. Black: events detected at HSPB or SPITS using classic waveform cross-correlation. Red: events detected at SPITS with array cross-correlation [13].

2.3 Interpretation

The magnitude-frequency distribution does not follow a classic Gutenberg-Richter law (power-law distributed seismic amplitudes) which results in an abnormally high exponent of 2.9 (Fig. 3, Main Text). Earthquake swarms in volcanic regions often experience such high exponents (b-value) suggested to reflect the lower stress [14] due to the presence of magmatic fluids that weaken the crust [8, 9]. This is analogous with the progression of the Zawadzkibreen surge bulge that weakened and damaged the ice left behind the bulge and along the lateral edges of the glacier. High resolution imagery (Google Earth, July 7, 2009) after the collapse reveal rifts (0.5 and 1 km) at the confluences of two smaller tributary cirques into Zawadzkibreen. The rifts coincide with upper limit of two intensely and transversely crevassed ice regions with chaotic, square-top blocky and rhombic patterns [15] as a result of longitudinal extension. The presence of crevasses along this lateral edge in 2008 and a V shaped pattern of diagonally striped positive and negative elevation changes prior to collapse (2003-2008) suggest ice was significantly weakened and under immense strain with press from above and restrain from the frozen valley sides and tongue below.

To test source region sizes, we calculate the seismic moment (M_0). The overall observed seismic moment release on the order of 10^{14} Nm corresponds to a rather small source area (10s of meters) with a slip distances less than 50 m. Since the event catalog is limited

to magnitudes greater than 0.5, the observed seismicity is only a small fraction of the (potential) total energy release and thus M_0 is a minimum estimate. Rifts here (0.5-1.5 km long) would produce a theoretical $M0$ of 10^{17} Nm if they were 100-200 meters deep and with displacements of up to a kilometer. The same slip area results in 10^{14} Nm if the total slip displacements would be only on the meter magnitude.

3 Remote Sensing Analysis

3.1 Radar backscatter

To reconstruct the onset and evolution of the NGS at high temporal resolution, we used radar backscatter intensity from daily and sub-daily Envisat ASAR C-band wide-swath mode scenes, collected from the 1st of January 2007 to the 31st of December 2010 (Fig. S3). Backscatter follows a seasonal cycle with low values in summer when snow and ice surfaces are wet and generally higher values in winter corresponding to colder snow and firn surfaces. However, rough surfaces, such as intensely crevassed areas in the ablation zone, also result in high backscatter intensity. Training sets in highly crevassed areas corresponded to a backscatter greater than – 5 dB, so this value was chosen to discriminate between chaotically crevassed ice (> -5 dB) and smoother glacier ice (< -5 dB). A mask of 225 km^2 following the outlines of the lower part of the glacier was used to clip the scenes (see Fig. S3). The mask accommodates for maximum advance that the glacier reached in 2010, but avoids the accumulation area of the NGS. The number of pixels was then converted to area. Some days are covered with more than one scene, we computed daily averaged and display a moving average of 5 days.

3.2 Geometric Observations and Interpretations

In the following, we present additional details from our remote sensing analysis. Between 1936 and 1961, the Nathorst glacier system rapidly retreated 5-6 km (200-240 ± 5 m a^{-1}) through the deepest part of the fjord . Retreat rates reduced through the 70's and 80s (70-100 ± 2 m a^{-1}) to about 50 ± 3 m a^{-1} in 90s. By 1996, velocities at the terminus were

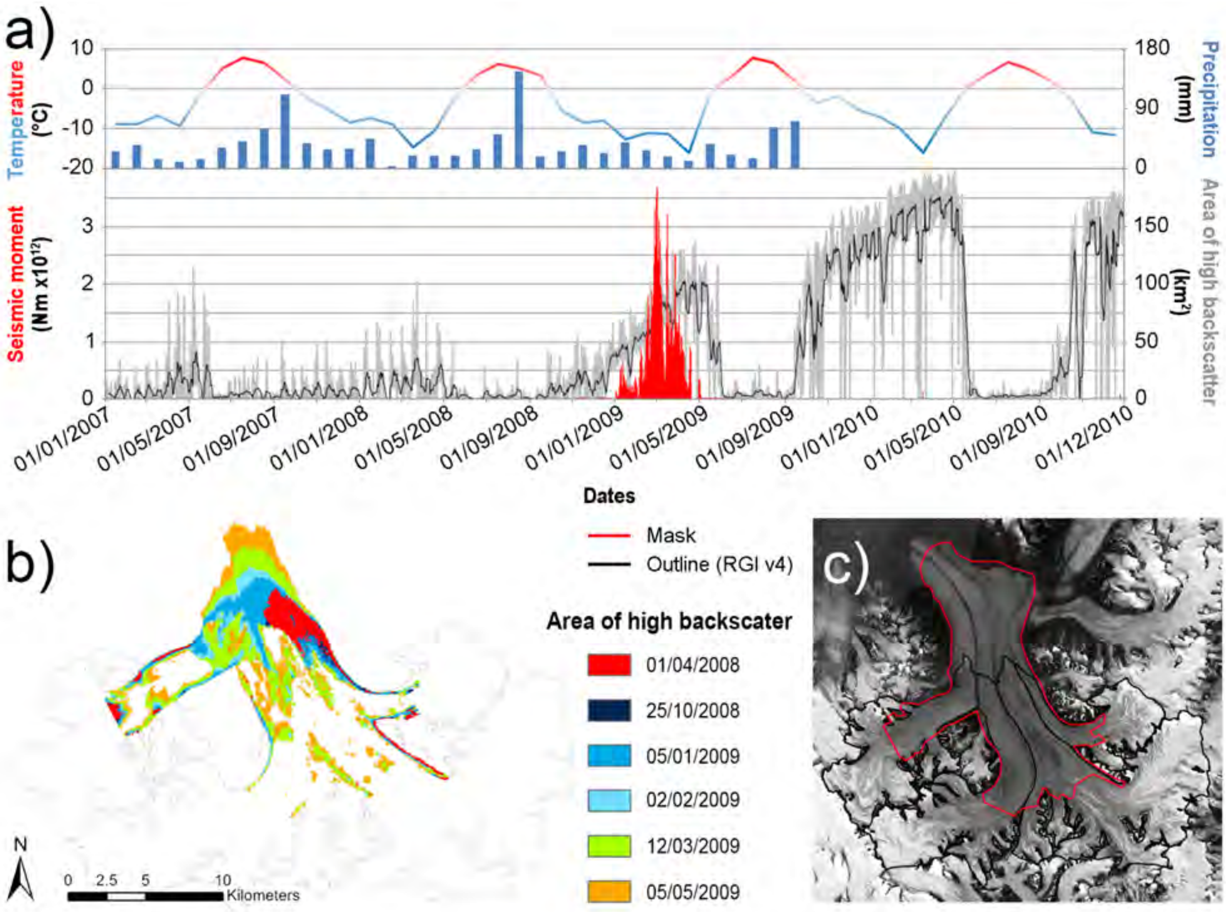

Figure S3: **Classification of radar backscatter through time** a) Lower panel: evolution of the total area of high backscatter (> -5 dB interpreted as intense crevassing) throughout the surge. Daily averages are plotted in gray, and a 5-day moving average is plotted in black. Cumulative seismic moment release per day is plotted against the area of high backscatter. Upper panel: temperatures (from Longyearbyen airport) and precipitation (from Horsund station). Precipitation record is discontinued after September 2009. b) Spatial distribution of the areas of high backscatter before, during and after detection of the seismic events. c) Map of the NGS with outlines (in black) from the RGI v4, and mask used to clip the Envisat scenes for classification. Background image from summer 2011.

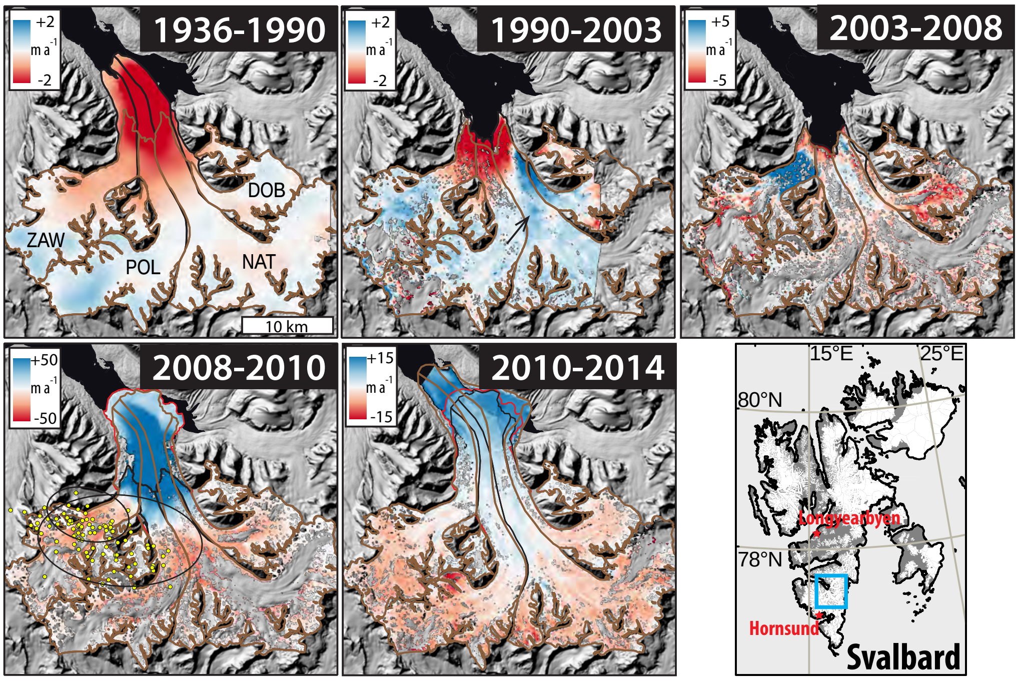

Figure S4: Elevation changes of the Nathorst Glacier System (NGS) and the 4 main tributaries for five epochs covering the past 78 years. Note the different color scale range for the different epochs. The black arrow (1990-2003) shows the location of the anomalous elevation change pattern interpreted as growth of a subglacial lake and sediment storage location. The 98 located seismic events are shown as yellow dots with black error ellipses (2008-2010).

stagnant, not more than a centimeter per day. Further upstream, velocities gradually increased with the maximum of 6-7 centimeters per day experienced on Dobrowolskibreen (Fig. S5). Between 1990 and 2003, Dobrowolskibreen showed signs of surging with thickening (2-3 m a^{-1}) in the ablation area and thinning above 400 masl (Fig. 3). Moreover, horizontal velocities of a maximum 1996/97 velocities of the entire NGS catchement are observed here with 0.06 m d^{-1} in the upper areas (Fig. S5). In 2003, there was a 40-50 m high surge bulge at the confluence with Nathorstbreen and a 10-20 m thick forebulge extending radially outwards 1 km from the medial moraine. Interestingly, the moraine was not horizontally displaced in orthoimages from 1990 and 2003 suggesting thickening on the Nathorstbreen side of the medial moraine results from basal emergence, ice and/or sediments. The terminal moraine of Dobrowolskibreen where it wedged between the Nathorstbreen tongue and the valley side began migrating in 2000, about 400 m by 2003 (0.3 m day^{-1}), another 500 m by 2005 (0.7 m day^{-1}), and eventually reaching the calving front by 2008 with speeds of 3-5 m d^{-1} (Fig. S6). The appearance of a thin swath of surface crevasses in 2005 along the forebulge up glacier denote the shear margin of the fast flow region that incorporated parts of the Ljosfonn terminus in the August 2008 velocity field. In this case, the surge bulge was not large enough to mechanically break the frozen terminus.

Meanwhile on the other side of NGS, the tributary Zawadzkibreen started surging between 1990 and 2003, slightly delayed to the Dobrowolskibreen surge. The 1996/97 horizontal velocities suggest horizontal migration of the surge bulge began after this point, with the lowest velocities of the entire NGS catchment (Fig. S5). The frozen terminus continued thinning (3 m a^{-1}) and a 10-20 m thick bulge had formed 6-8 km upstream by 2003 (Fig. 3). The bulge spanned the 2.5 km width of Zawadzkibreen and progressed down-glacier at speeds greater than 2 ± 0.5 m d^{-1} through 2006. A few months before the collapse in 2008, the bulge had slowed (1.7 m d^{-1}) and thickened immensely as a consequence of the enhanced strength provided by the frozen glacier tongue. The bulge was then 50 m high and pressing the medial moraine at the confluence with Polakkbreen. Horizontal velocities (Aug 4 to Sept 1, 2008) on the bulge were about 1 m d^{-1} while the

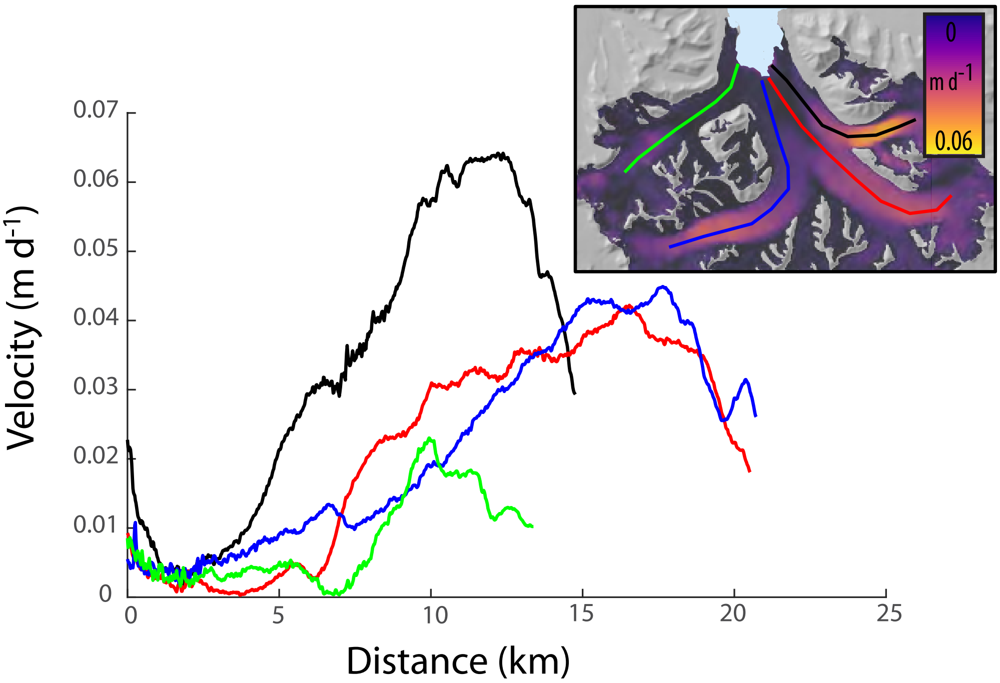

Figure S5: Centerline velocities from the 1996/96 ERS 1/2 radar images for the four tributaries: Doborowolskibreen (black), Ljosfonn (red), Polakkbreen (blue), and Zawadzkibreen (green).

glacier tongue was still strongly held to the bed except a narrow path moving at maximum 0.5 m d^{-1} (Fig. S6). This swath of slightly higher velocities may be a newly formed basal discharge channel or development of a new shear margin.

A peculiar circular pattern of elevation increases seen between 1990 and 2003 (main text, Fig. 2) resembles surface expressions caused from subglacial lake drainage and refilling [16]. In 2003, a 20 m high surface bump (1 km diameter) is visible downstream from a bedrock bump [17]) in a depression common on the lee sides [18]. Basal water and sediments from the Dobrowolskibreen surge likely spilled under Nathorstbreen over years collecting in this bedrock depression. Elevation changes from 2003 to 2008 show thinning above the 2003 surface bump and thickening below expressing an internal surge. In August 2008, a new small surge bulge outlines the frozen ice transition 1 km from the calving front with horizontal velocities up to 1 m d^{-1} (Fig. S6). These chain of events seem likely caused by the excess water and sediments from the surge of a small tributary glacier.

After the collapse, we are able to derive velocities of NGS for the following 4 years

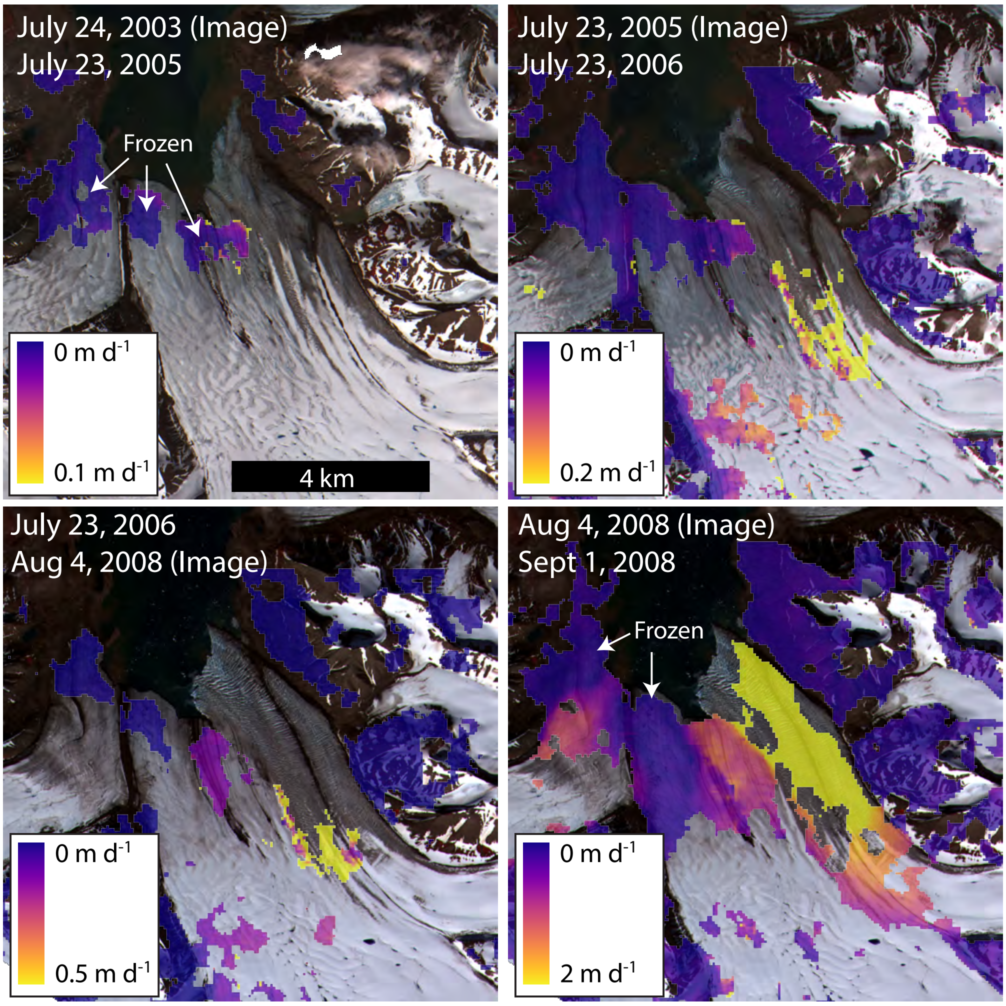

Figure S6: Displacements and glacier velocities measured from repeat ASTER and SPOT optical images. Only high correlation pixels ($> 70\%$) are shown. The lowest magnitude displacements reveals the frozen, stagnant NGS glacier tongue. Note the different color scale range for the different epochs. The August to September 2008 displacements reveal processes occurring a few months prior to collapse.

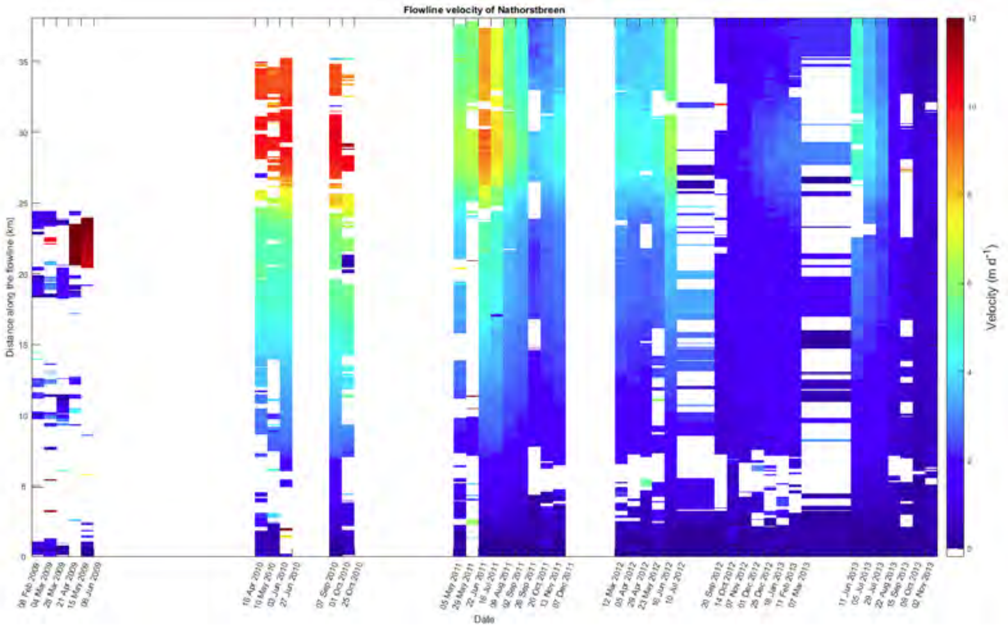

Figure S7: **Velocity along the centerline of Ljosfonn** Distance begins up-glacier with the largest distance at the glacier terminus. Color ramp is saturated above 12 meters per day to highlight the immediate post collapse velocity.

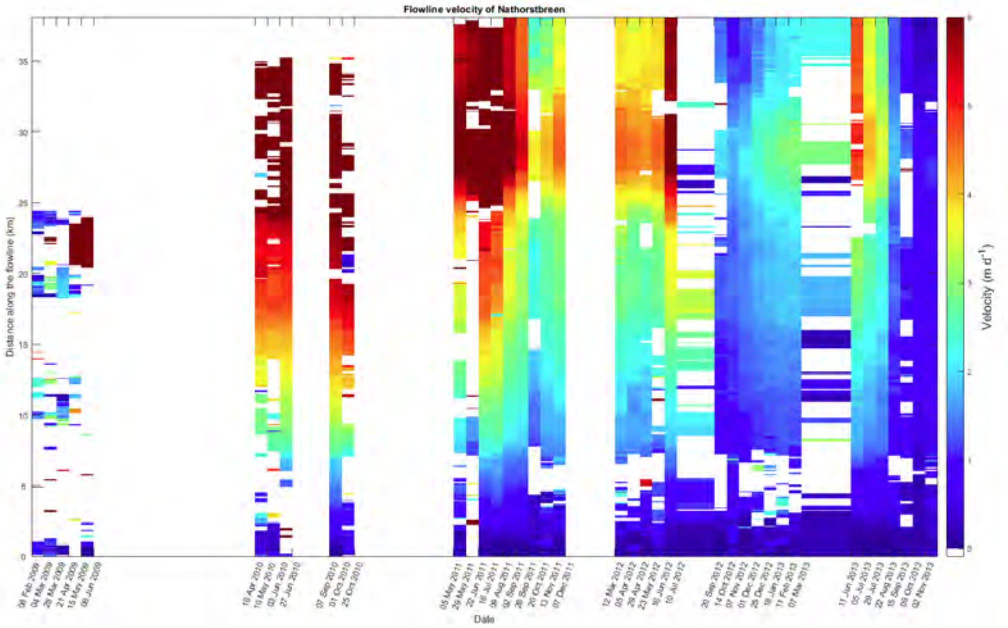

Figure S8: **Velocity along the centerline of Ljosfonn** Distance begins up-glacier with the largest distance at the glacier terminus. Color ramp is saturated above 6 meters per day to highlight smaller changes post collapse.

by feature tracking on Radarsat-2 imagery collected every 24 days (Fig. S7 and S8). Velocities through 2010 were above 10 m d^{-1} on the advancing tongue which by summer 2011 drops to 6-8 m d^{-1}. At this time, the glacier experienced a summer speed up (max. 8-9 m d^{-1}), a more typical dynamic response related to melt water variation. In winter 2011-2012, velocities over the entire glacier reduced to 2-3 m d^{-1}.

The total volume changes and geodetic mass balances are shown in Tables S2 and S3. Glacier volume change of the entire NGS system during the 2008-2010 period containing the collapse was 500-700% larger than the long term average. Interestingly, the average rate of volume change of the Polakkbreen and Zawadzkibreen catchments are slightly larger in the years after collapse. Doborowolskibreen, on the other hand, had close to zero volume change on average during the four years post collapse. Further analysis on the mass changes of the glacier during and after the collapse are described in the next section and Fig. S14.

The sequence of events observed for Nathorstbreen is remarkably analogous to the surges of the Paulabreen-Bakaninbreen catchment (a geometrically similar glacier, 50 km north of NGS). First, Bakaninbreen surged in the late 1980s though never progressed to the terminus (Dobrowolskibreen analogue). Sediment intrusions along thrust faults observed on the forebulge [19, 20] suggest frozen conditions of the soft-bed provided enough friction to withstand the driving stress of the surge bulge [21]. On the other side of Paulabreen, the 2003 surge of Skobreen pressed the medial moraine (Zawadzkibreen analogue) and advanced the terminus [22]. However, the Paulabreen catchment did not collapse as did the NGS, possibly due to larger friction provided by the thermal and frictional geometry of the tongue with respect to the timing of individual tributary instabilities.

Table S2: Glacier volume change in km^3 yr^{-1} of the Nathorst Glacier System (NGS) and its four tributaries. Errors relate to 1σ.

**Time**	**Dobrowolskibreen**		**Zawadzkibreen**		**Polakkbreen**		**Ljosfonn**		**NGS**	
1936-1990	-0.005	±0.004	-0.045	±0.007	-0.026	±0.006	-0.058	±0.008	-0.134	±0.013
1990-2003	-0.003	±0.005	0.014	±0.008	0.012	±0.008	0.047	±0.012	0.071	±0.017
2003-2008	-0.066	±0.012	-0.016	±0.018	-0.050	±0.017	-0.190	±0.025	-0.322	±0.037
2008-2010	-0.147	±0.014	-0.165	±0.040	-0.316	±0.037	-0.446	±0.030	-1.075	±0.064
2010-2014	-0.010	±0.015	-0.212	±0.045	-0.338	±0.069	-0.203	±0.053	-0.763	±0.099

Table S3: Geodetic Balance in m w.eq. yr $^{-1}$ of the Nathorst Glacier System (NGS) and its four tributaries. A conversion density factor of 0.85 was assumed. Errors relate to 1σ.

**Time**	**Dobrowolskibreen**		**Zawadzkibreen**		**Polakkbreen**		**Ljosfonn**		**NGS**	
1936-1990	-0.08	±0.08	-0.35	±0.07	-0.17	±0.05	-0.28	±0.04	-0.31	±0.06
1990-2003	-0.05	±0.11	0.12	±0.08	0.09	±0.07	0.24	±0.07	0.16	±0.08
2003-2008	-1.14	±0.25	-0.14	±0.18	-0.36	±0.15	-0.98	±0.15	-0.75	±0.18
2008-2010	-2.31	±0.27	-1.32	±0.38	-2.16	±0.29	-2.16	±0.17	-2.33	±0.28
2010-2014	-0.15	±0.26	-1.51	±0.38	-2.11	±0.51	-0.89	±0.27	-1.50	±0.35

4 Glacier Evolution Modelling

4.1 Model Parameters and Methods figures

Table S4: Parameters of the mass balance model using Longyearbyen Airport meteorological station daily time-series.

Name	Symbol	Values	Units	Sources
Degree day factor for snow	f_{snow}	$1.9\ 10^{-3}$	m w.eq. Day^{-1} K^{-1}	Tuned[1]
Degree day factor for ice	f_{ice}	$7.3\ 10^{-3}$	m w.eq. Day^{-1} K^{-1}	Tuned[1]
Precipitation Lapse Rate	$\frac{\mathrm{d}p}{\mathrm{d}z}$	230	% km^{-1}	Tuned[2]
Precipitation at sea level	p_{ref}	540	mm yr^{-1}	Fixed[3]
Temperature Lapse Rate	$\frac{\mathrm{d}T}{\mathrm{d}z}$	$6.0\ 10^{-3}$	K m^{-1}	Fixed[4]
Snow/rain threshold	T_{snow}	273.15	K	Fixed
Melting threshold	T_m	273.15	K	Fixed
Super-imposed ice factor	f_r	0.15	-	Fixed[5]

[1] Tuned on geodetic mass balance measurement over the period 1990-2003
[2] Tuned to fit snow accumulation estimated in Hagen et al.[23], in good accordance with Aas et al.[24]
[3] Aas et al.[24]
[4] Przybylak et al.[25]
[5] Gilbert et al.[26]

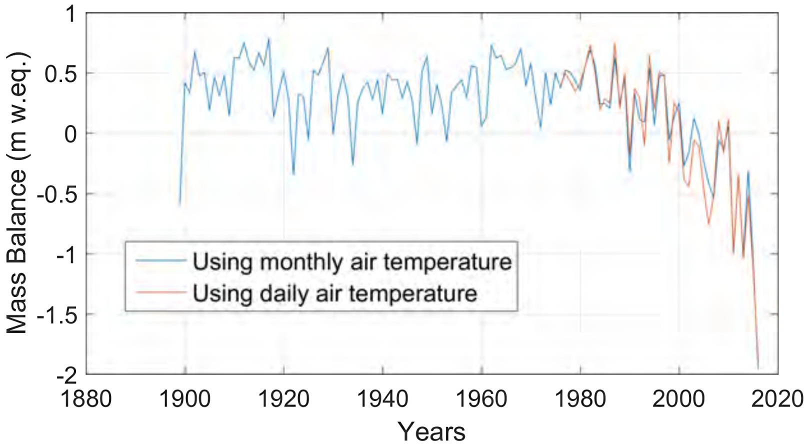

Figure S9: Modeled mean surface mass balance on NGS using daily (orange line) and monthly (blue line) air temperature from Longyearbyen Airport meteorological station

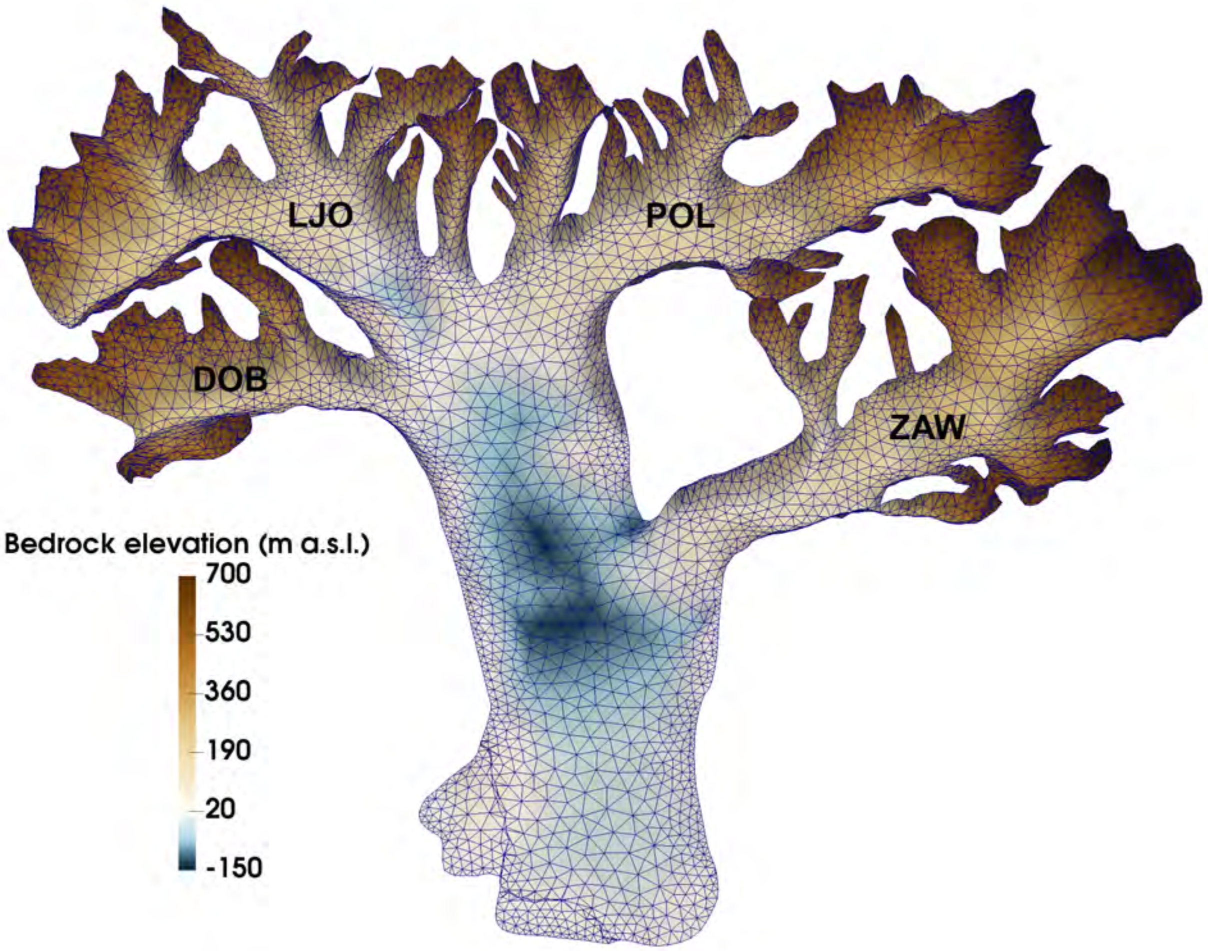

Figure S10: Basal topography and mesh used in the 3D model.

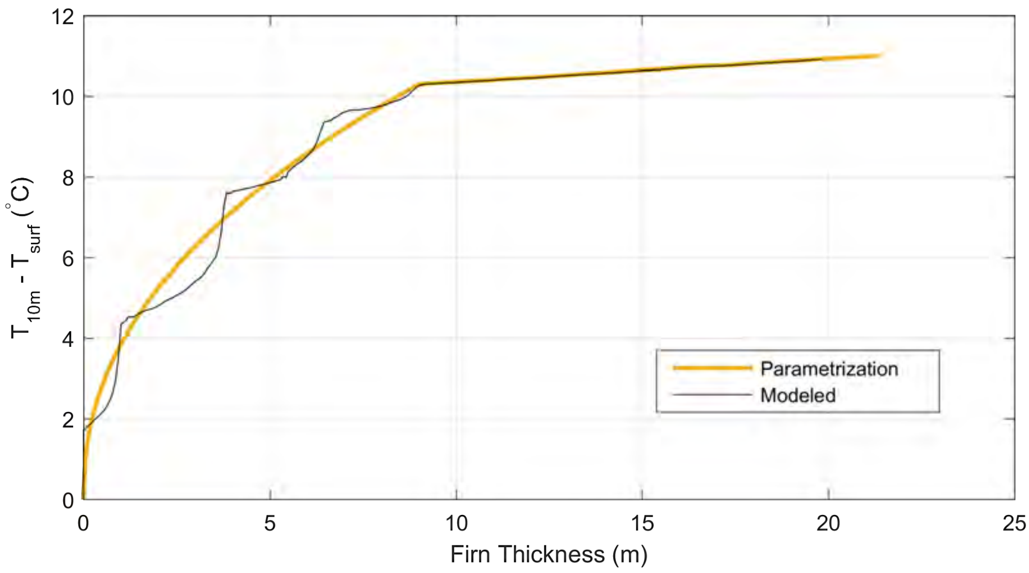

Figure S11: Modeled temperature difference between surface temperature and 10m-depth temperature as a function of firn thickness (black line) and associated fit (yellow line).

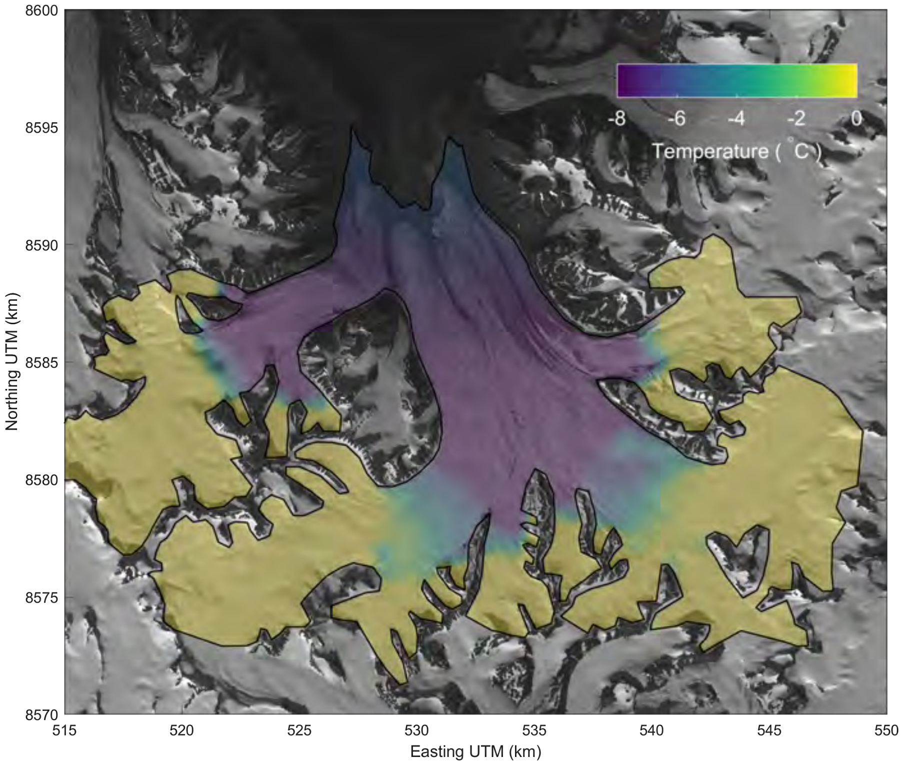

Figure S12: Steady state surface boundary condition for enthalpy computed from mean firn thickness and surface temperature over the period 1899-1960.

4.2 Surface mass balance

Modelled mass balance is in good accordance with a previous study [23] showing a mean equilibrium line altitude located around 300 m a.s.l. over the period 1974-2003 (Fig. S13). The modelled temporal evolution highlight a switch from a positive to a negative mean surface balance occurred in 2000 in response air temperature warming (Fig. S14). This corresponds to a large decrease in the size of the accumulation area (Fig. S15).

In order to estimate the influence of the hypsometry change linked to the 2008 surge on the surface mass balance, we computed the mean surface mass balance on the 2008 and 2014 geometry for the same climate (the 2000-2016 average). This shows that the NGS collapse led to a decrease of 35 % of the accumulation area associated with a decrease of 84% of the surface mass balance (Fig. S16). This change acting on an already unbalanced state means that the glacier will not be able to recover from the recent surge in the current climatic conditions.

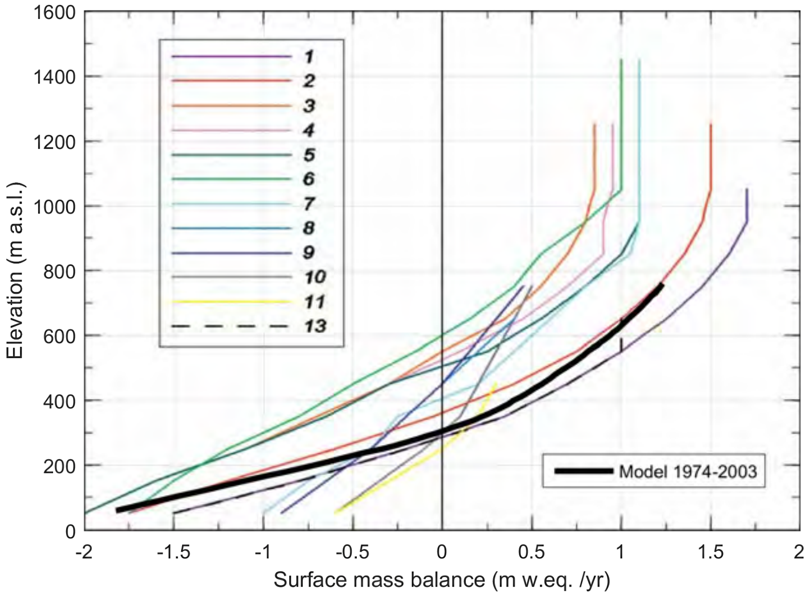

Figure S13: Modeled mean surface mass balance as a function of elevation compared to measurements [23]. Adapted from Figure 5 of Hagen et al. [23] with our model results as a thick black line.

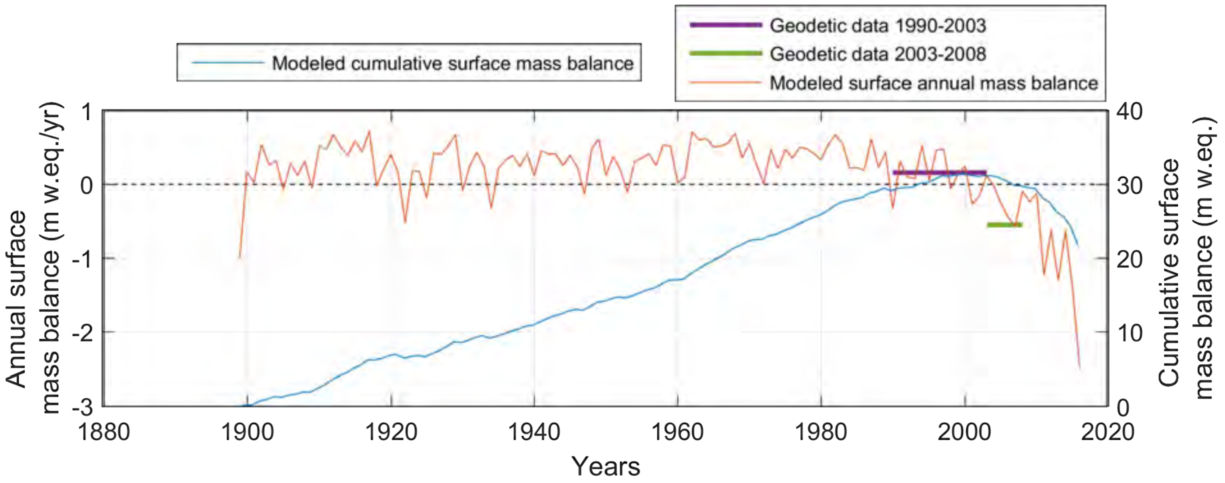

Figure S14: Modeled cumulative and annual surface mass balance on NGS. Also shown are geodetic mass balance estimates from 1990-2003-2008.

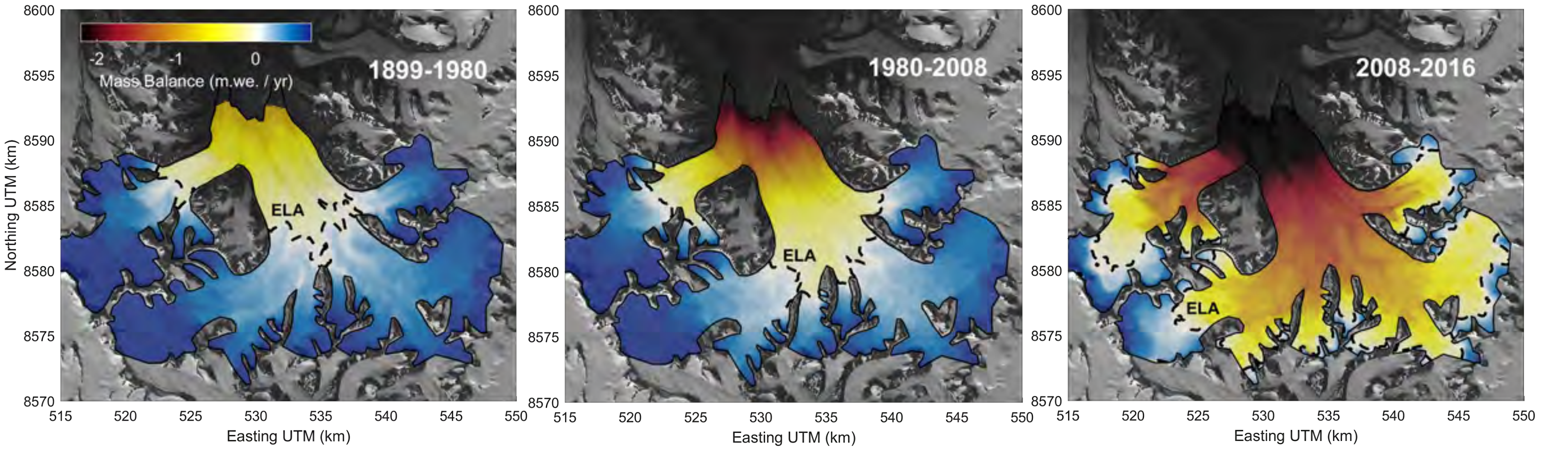

Figure S15: Modeled mean surface mass balance on NGS over the period 1899-1980, 1980-2008 and 2008-2016.

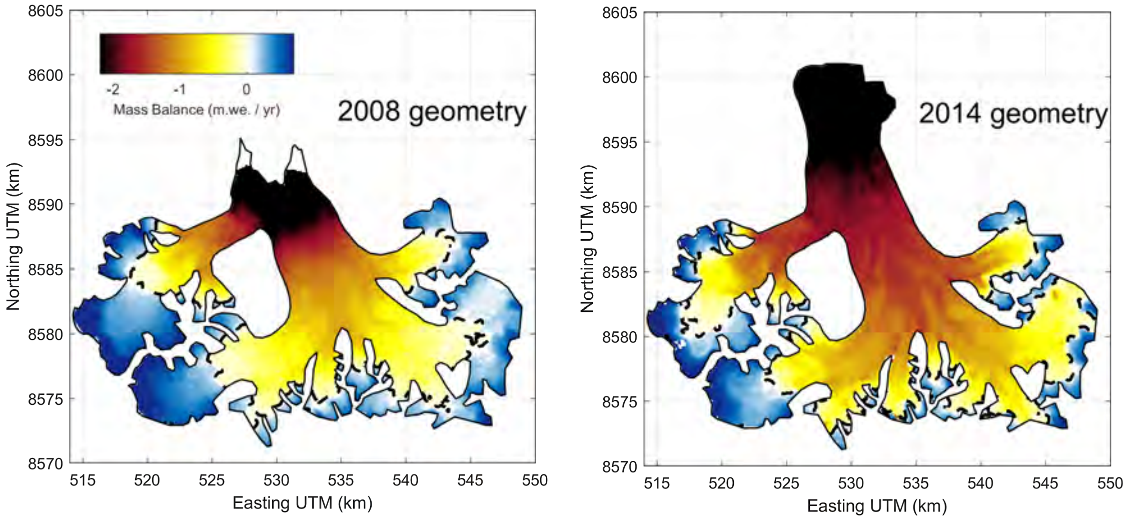

Figure S16: Modeled mean surface mass balance on NGS over the period 2000-2016 for pre-surge (2008) and post-surge geometry (2014).

4.3 Thermal regime and glacier geometry

In this section, we describe results from modeling the 3D thermal structure in the glacier and particularly at the base of the glacier between 1936 and 2008. In order to obtain an initial state in 1936 for the transient simulation, we adopted the following strategy:

1. We computed the steady state thermal structure with measured 1990 geometry and uniform basal friction.

2. We performed a transient simulation starting with the steady state thermal structure and applied a sudden decrease of the basal friction until the observed length in 1936 is reached.

3. We interpolated the modelled temperature field from our surging glacier to the real glacier geometry measured in 2010. We preferred to use the 2010 surging geometry rather than 1936 geometry on this profile as it is nearly identical to the 1936 geometric profile in the ablation area and is much more reliable in the accumulation area.

Table S5: Parameters of the thermo-mechanical model.

**Name**	**Symbol**	**Values**	**Units**
Basal friction during quiescent phase	β_q	See Fig. S17	M Pa yr m^{-1}
Basal friction during surge phase	β_s	10^{-5}	M Pa yr m^{-1}
Densification rate	a	1.4 10^{-2}	yr^{-1}
Maximum water content	ω_{max}	0.03	
Termperature ice moisture diffusivity	κ_0	1.045 10^{-4}	kg m^{-1}s^{-1}
Snow density	ρ_0	350	kg m^{-3}
Basal heat flux	F_b	0.040[1]	W m^{-2}

[1] Measured at Austfonna Ice Cap [27].

The glacier state in 1936 is the result from a surge a few decades earlier[28] and therefore the initial glacier state is obtained by simulating a surge event starting from a steady state glacier. Further, the initial steady state glacier was computed using a constant climatic condition corresponding to a steady mass balance for the pre-surge topography measured in 1990. We assume that calving flux is negligible during all simulations and simply assume that ice is removed when the surface elevation reaches 50 m which allows the

maintenance of a realistic tidewater glacier geometry. Simulations were performed for the parameter set summarized in table 4.3. Transient simulation starting in 1936 and forced by the calibrated mass balance model were run to estimate temperature evolution between 1936 and 2008. We compared three alternate friction fields: (i) uniform friction coefficient, (ii) spatially variable friction coefficient inferred from the 1996/97 velocity field, and (iii) a binary distribution with a constant high friction on the tongue, inferred from the 1996/97 velocity field, and uniform low friction (more sliding) elsewhere (see Fig. S17).

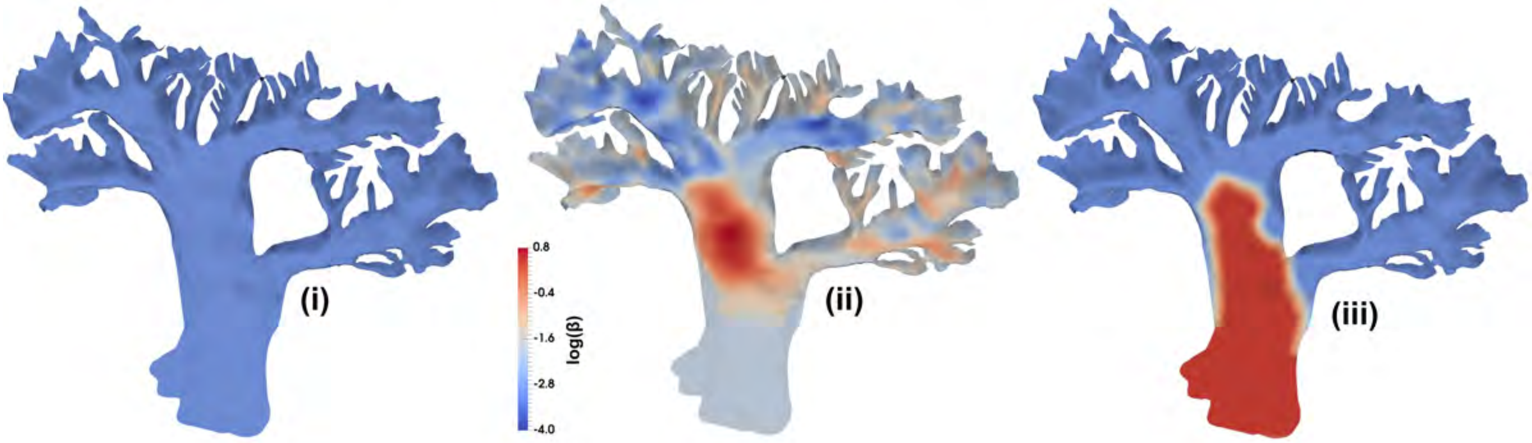

Figure S17: Different friction coefficient assumption during the transient run between 1936 and 2008. (i) Uniform friction, (ii) inverted friction in 1996 and (iii) parametrized friction with a transition threshold between high and lower friction

Results show that the steady state thermal regime of NGS is characterized by a temperate accumulation area and a surface cold ice layer that partially reaches the base of the glacier in the tongue region (Fig. S18). These frozen regions occur first at the convergence zones of individual streams of ice where ice advection is lowest (Fig. S21). The surge event enlarges the temperate areas (Fig. S19) that are then able to refreeze during quiescent phase within some decades.

The uncertainty linked to these results mainly rely on the absence of calving model, the assumption of constant basal friction coefficient during time and the uncertainty of the initial state in 1936. However, our observations show that ice is immobile in the tongue region, and likely during the whole quiescent phase which limits calving activity. The influence of the friction hypothesis between 1936 and 2008 leads to differing glacier

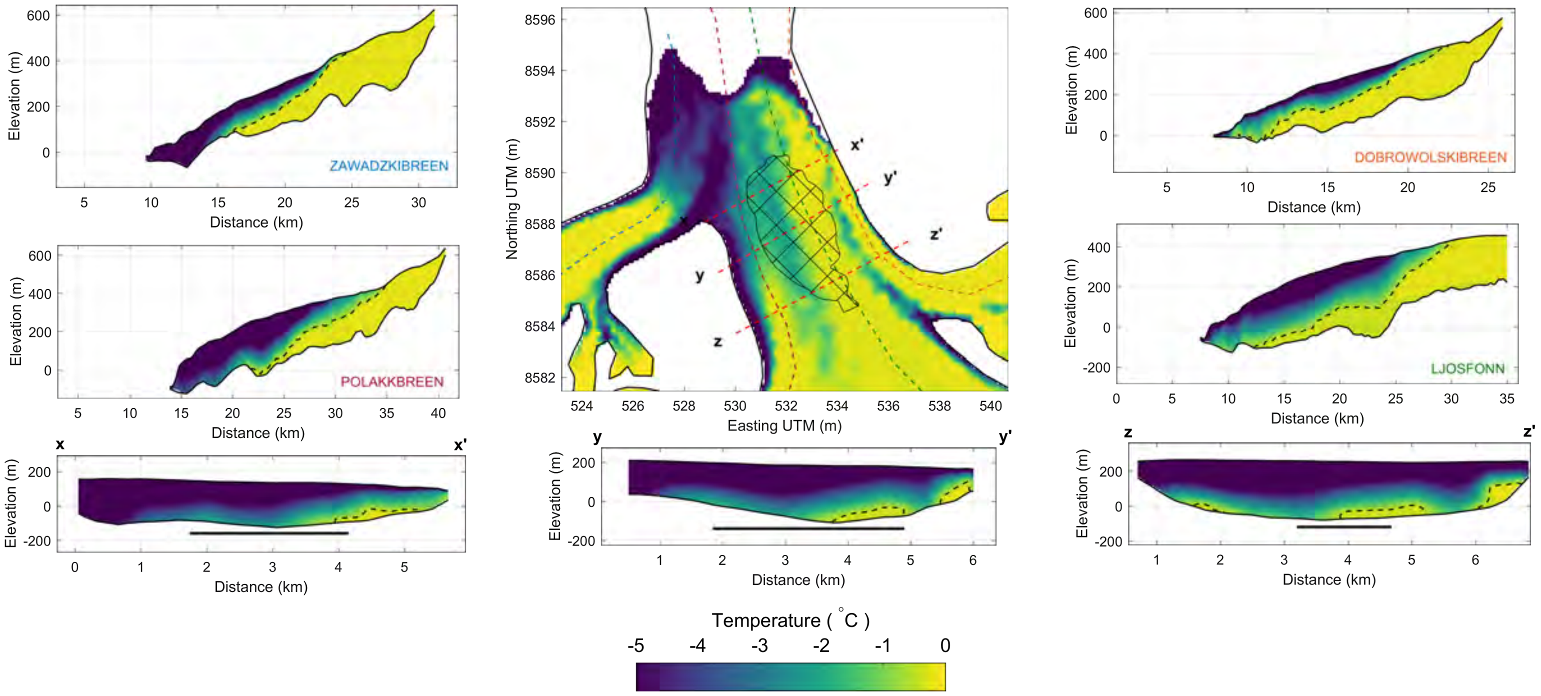

Figure S18: Steady state temperature computed with 1990 surface topography and inverted basal friction from InSAR velocities.

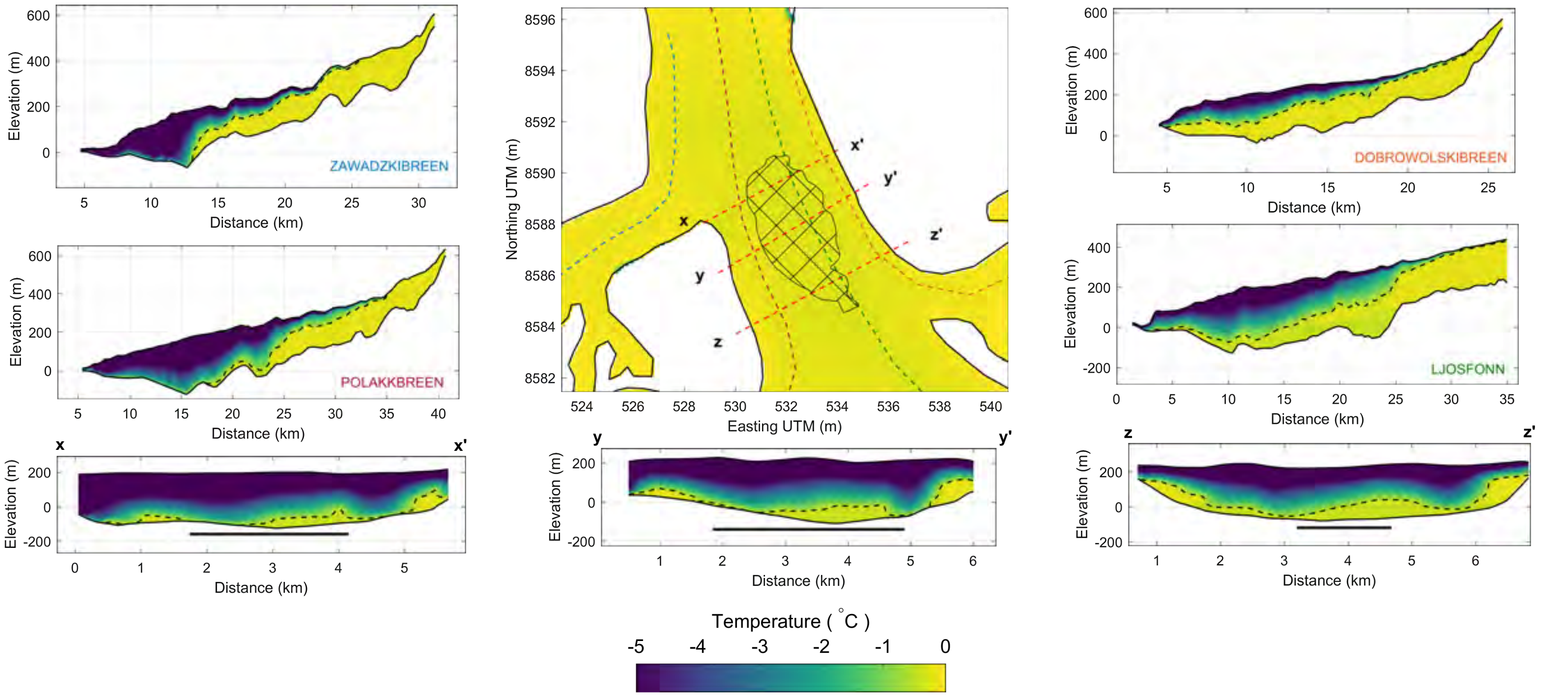

Figure S19: Modeled temperature for initial state in 1936 after surge.

geometries though with similar thermal structure (Fig. S20), increasing confidence in our modelled temperature field. However, observed surface topography in 1990 can only be reproduce assuming (iii) suggesting high basal friction of the tongue was already established by 1936. Importantly, our modelled Cold-Temperate transition surface (CTS) agrees remarkably with that measured from radio echo sounding along the Ljosfonn branch [17] proving the reliability of both initial state simulations and transient runs.

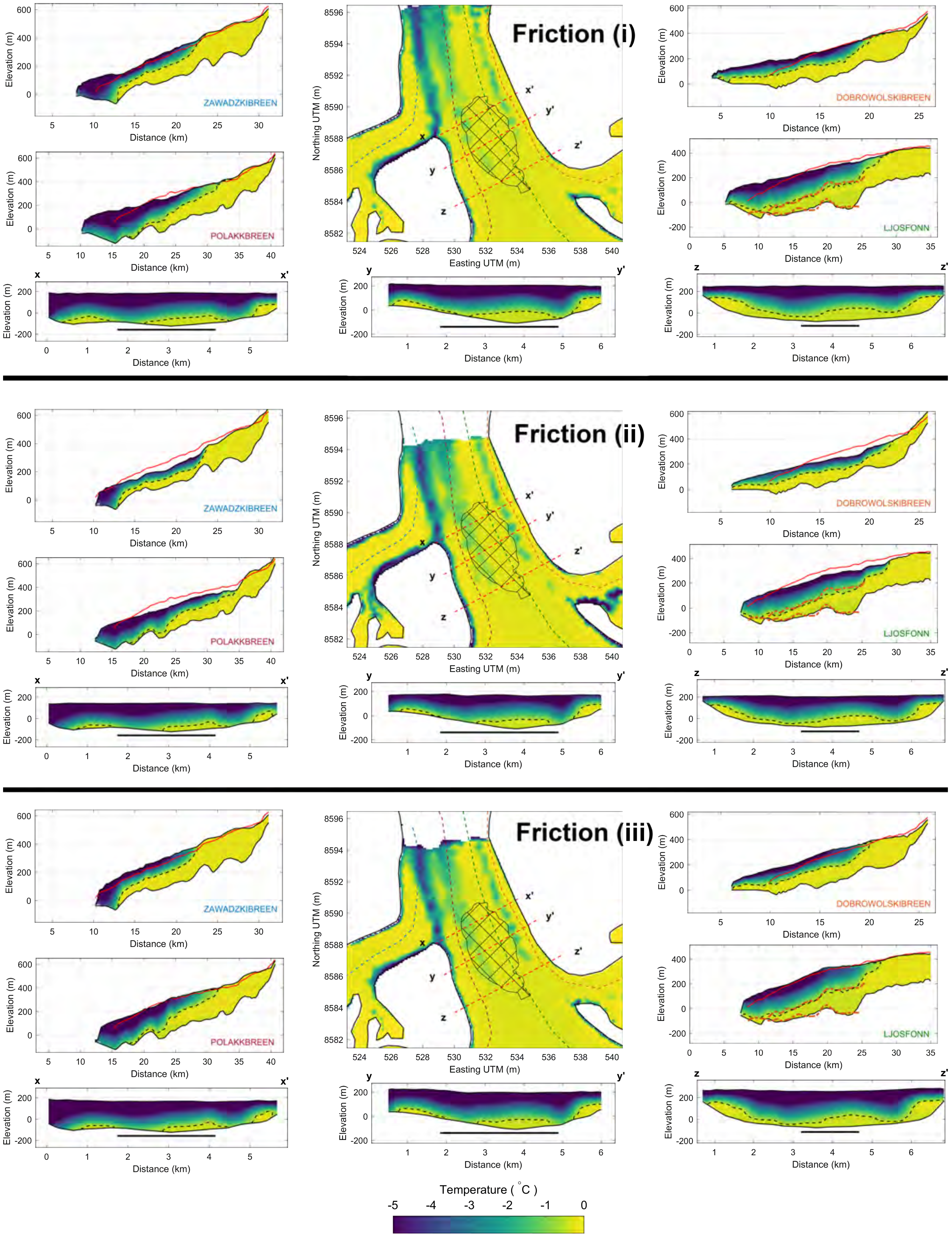

Figure S20: Modeled temperature and glacier geometry in 1990 for the three different friction hypothesis. Red lines are measured surface topography in 1990. Orange dashed line on Ljosfonn panels are measured bed topography and Cold-Temperate Surface transition [17]

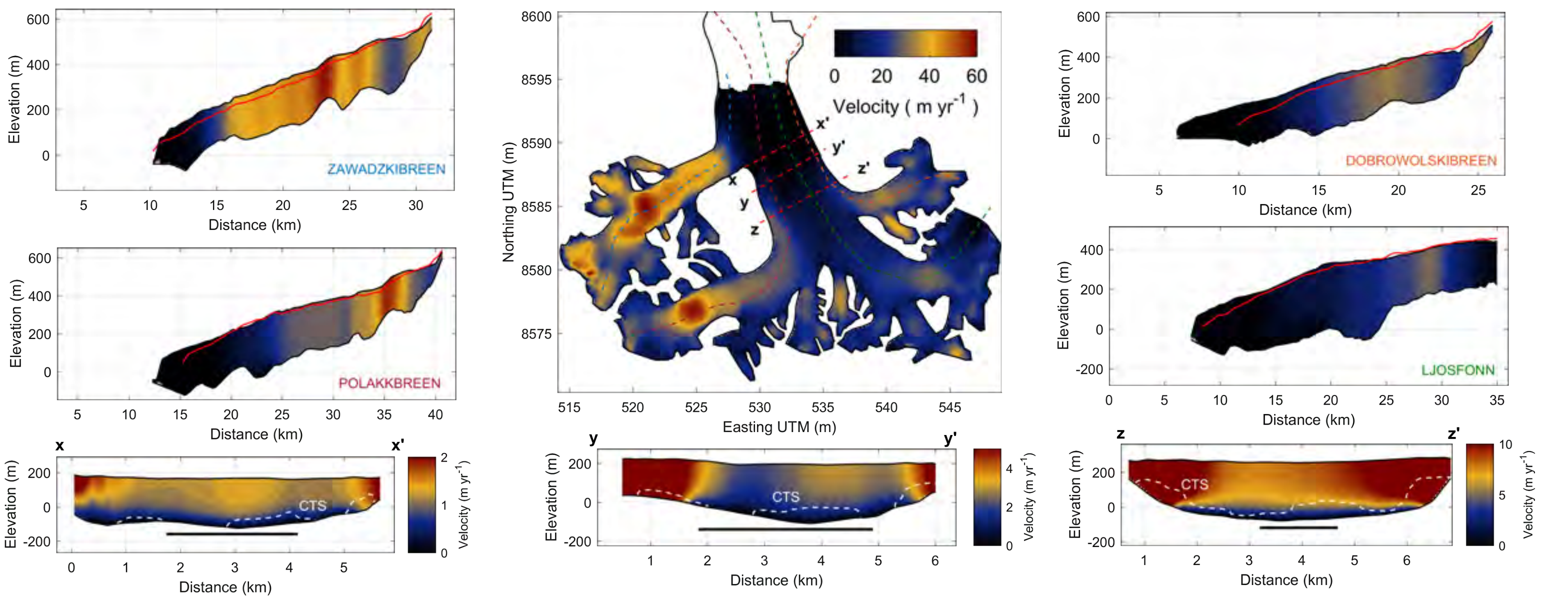

Figure S21: Modeled velocity magnitude in 1990 for friction hypothesis (iii). Modeled cold-Temperate Surface (CTS) is indicated with dashed white line on the transverse profiles

References

1. Girod, L., Nuth, C. & Kääb, A. Improvement of DEM generation from ASTER images using satellite jitter estimation and open source implementation. *Int. Arch. Photogramm. Remote Sens. Spatial Inf. Sci.* **XL-1/W5,** 249–253 (2015).

2. Korona, J., Berthier, E., Bernard, M., Rémy, F. & Thouvenot, E. SPIRIT. SPOT 5 stereoscopic survey of Polar Ice: Reference Images and Topographies during the fourth International Polar Year (2007-2009). *ISPRS Journal of Photogrammetry and Remote Sensing* **64,** 204–212 (2009).

3. Moreira, A. *et al. TanDEM-X: a TerraSAR-X add-on satellite for single-pass SAR interferometry* in. **2** (IEEE, 2004), 1000–1003.

4. Schellenberger, T., Van Wychen, W., Copland, L., Kääb, A. & Gray, L. An Inter-Comparison of Techniques for Determining Velocities of Maritime Arctic Glaciers, Svalbard, Using Radarsat-2 Wide Fine Mode Data. *Remote Sensing* **8** (2016).

5. Girod, L., Nuth, C., Kääb, A., McNabb, R. & Galland, O. MMASTER: Improved ASTER DEMs for Elevation Change Monitoring. *Remote Sensing* **9** (2017).

6. Pirli, M., Schweitzer, J. & Paulsen, B. The Storfjorden, Svalbard, 2008-2012 after-shock sequence: Seismotectonics in a polar environment. **601,** 192–205 (2013).

7. Alstott, J., Bullmore, E. & Plenz, D. powerlaw: a Python package for analysis of heavy-tailed distributions. *PloS one* **9,** e85777 (2014).

8. Farrell, J., Husen, S. & Smith, R. B. Earthquake swarm and b-value characterization of the Yellowstone volcano-tectonic system. *The Track of the Yellowstone HotspotWhat do Neotectonics, Climate Indicators, Volcanism, and Petrogenesis Reveal about Subsurface Processes?* **188,** 260–276 (Nov. 2009).

9. McNutt, S. R. VOLCANIC SEISMOLOGY. *Annu. Rev. Earth Planet. Sci.* **33,** 461–491 (2005).

10. Podolskiy, E. A. & Walter, F. Cryoseismology. *Rev. Geophys.* **54,** 708–758 (2016).

11. Danesi, S., Bannister, S. & Morelli, A. Repeating earthquakes from rupture of an asperity under an Antarctic outlet glacier. *Earth* **253,** 151–158 (2007).

12. Zoet, L. K., Anandakrishnan, S., Alley, R. B., Nyblade, A. A. & Wiens, D. A. Motion of an Antarctic glacier by repeated tidally modulated earthquakes. *Nature Geosci* **5,** 623–626 (2012).

13. Gibbons, S. J. & Ringdal, F. The detection of low magnitude seismic events using array-based waveform correlation. *Geophys. J. Int.* **165,** 149–166 (2006).

14. Schorlemmer, D., Wiemer, S. & Wyss, M. Variations in earthquake-size distribution across different stress regimes. *Nature* **437,** 539–542 (Sept. 2005).

15. Herzfeld, U., Clarke, G., Mayer, H. & Greve, R. Derivation of deformation characteristics in fast-moving glaciers. *Computers & Geosciences* **30,** 291–302 (2004).

16. Willis, M. J., Herried, B. G., Bevis, M. G. & Bell, R. E. Recharge of a subglacial lake by surface meltwater in northeast Greenland. *Nature* **518,** 223–227 (2015).

17. Dowdeswell, J. A., Drewry, D. J., Liestol, O. & Orheim, O. Airborne radio echo sounding of sub-polar glaciers in Spitsbergen. *Norwegian Polar Institute - Skrifter Nr. 182,* 41 (1984).

18. Iverson, N. R. Potential effects of subglacial water-pressure fluctuations on quarrying. *Journal of Glaciology* **37,** 27–36 (1991).

19. Porter, P. R., Murray, T. & Dowdeswell, J. A. Sediment deformation and basal dynamics beneath a glacier surge front: Bakaninbreen, Svalbard. *Annals of Glaciology* **24,** 21–26 (1997).

20. Murray, T. *et al.* Glacier surge propagation by thermal evolution at the bed. *Journal of Geophysical Research: Solid Earth* **105,** 13491–13507 (2000).

21. Stuart, G., Murray, T., Brisbourne, A., Styles, P. & Toon, S. Seismic emissions from a surging glacier: Bakaninbreen, Svalbard. *Annals of Glaciology* **42,** 151–157 (2005).

22. Kristensen, L. & Benn, D. A surge of the glaciers Skobreen-Paulabreen, Svalbard, observed by time-lapse photographs and remote sensing data. *Polar Research* **31,** 11106 (Jan. 2012).

23. Hagen, J. O., Melvold, K., Pinglot, F. & Dowdeswell, J. A. On the net mass balance of the glaciers and ice caps in Svalbard, Norwegian Arctic. *Arctic Antarctic and Alpine Research* **35,** 264–270 (2003).

24. Aas, K. S. *et al.* The climatic mass balance of Svalbard glaciers: a 10-year simulation with a coupled atmosphere–glacier mass balance model. *The Cryosphere* **10,** 1089–1104 (2016).

25. Przybylak, R. *et al.* Spatial distribution of air temperature on Svalbard during 1 year with campaign measurements. *International Journal of Climatology* **34,** 3702–3719 (2014).

26. Gilbert, A. *et al.* Sensitivity of Barnes Ice Cap, Baffin Island, Canada, to climate state and internal dynamics. *J. Geophys. Res. Earth Surf.* **121,** 1516–1539 (2016).

27. Zagorodnov, V., Savatyugin, L. & Morev, V. Temperature regime of the Akademiya Nauk Glacier, Severnaya Zemlya. *Data of Glaciological Studies* **65,** 134–138 (1989).

28. Ottesen, D. *et al.* Submarine landforms characteristic of glacier surges in two Spitsbergen fjords. *Quaternary Science Reviews* **27,** 1583 –1599 (2008).
